# Development and Validation of Machine Learning Models for Predicting Falls Among Hospitalized Older Adults: Retrospective Cross-Sectional Study

**DOI:** 10.2196/80602

**Published:** 2026-01-05

**Authors:** Xiyao Yang, Juan Ren, Dan Su, Manzhen Bao, Miao Zhang, Xiaoming Chen, Yanhua Li, Zonggui Wang, Xiujing Dai, Zengzeng Wei, Shuiyu Zhang, Yuxin Zhang, Juan Li, Xiaolin Li, Junjin Xu, Nan Mo

**Affiliations:** 1 Second Hospital of Anhui Medical University, No.678 Furong Road, Shushan District, Hefei, 230601, China, 86 1-809-650-2125; 2 School of Nursing, Anhui Medical University, Hefei, China; 3 The Taikang Health and Wellness Industry Research Institute, Anhui Medical University, Hefei, China

**Keywords:** machine learning, risk prediction, older adults, fall prediction, gradient boosting machine, random forest

## Abstract

**Background:**

Falls are one of the leading causes of injury or death among older adults. Falls occurring in individuals during hospitalization, as an adverse event, are a key concern for health care institutions. Identifying older adults at high risk of falls in clinical settings enables early interventions, thereby reducing the incidence of falls.

**Objective:**

This study aims to develop and validate machine learning models to predict the risk of falls among hospitalized older adults.

**Methods:**

This study retrospectively analyzed data from a tertiary general hospital in China, including 342 older adults who experienced falls and 684 randomly matched nonfallers, between January 2018 and December 2024, encompassing demographic information, comorbidities, laboratory parameters, and medication use, among other variables. The dataset was randomly split into training and testing sets in a 7:3 ratio. Predictors were selected from the training set using stepwise regression, least absolute shrinkage and selection operator, and random forest-recursive feature elimination. Seven machine learning algorithms were employed to develop predictive models in the training set, and their performance was compared in the testing set. The optimal model was interpreted using Shapley Additive Explanations (SHAP).

**Results:**

The gradient boosting machine model demonstrated the best predictive performance (C-index 0.744, 95% CI 0.688‐0.799). The 8 most important variables associated with fall risk were dizziness, epilepsy, fall history within the past 3 months, use of walking assistance, emergency admission, Morse Fall Scale scores, modified Barthel Index scores, and the number of indwelling catheters. The model was interpreted using SHAP to enhance the clinical utility of the predictive model.

**Conclusions:**

The gradient boosting machine model was identified as the optimal predictive model. The SHAP method enhanced its integration into clinical workflows.

## Introduction

Falls, the second leading cause of global unintentional injury deaths, are a significant public health concern. They are defined as “an event that leads to a person inadvertently coming to rest on the ground, floor, or other lower surface than their original position [1].” Age is one of the main risk factors for falls [2], and statistics indicate that the incidence of falls among older adults is approximately 26.5% [3]. Among individuals aged >60 years globally, falls are one of the most common causes of injury or death, with one out of every 5 falls resulting in a fracture or head injury [2,4]. In addition, falls generate substantial medical costs, imposing a heavy economic burden worldwide [5].

Notably, falls are adverse events in hospitals, and the prevention of falls is also a priority for improving the quality of nursing care [6]. The incidence of falls in hospitals is typically in the range of 2 to 16 per 1000 bed days [7,8]. Despite a declining incidence of falls among hospitalized older adults, the increasing number of older adults admitted to hospitals, driven by an expanding aging population, suggests that falls prevention will remain a critical concern in hospitals [8,9]. Falls are preventable adverse events in hospitals, and implementing fall prevention programs can avoid costs of US $14,600 per 1000 patient-days [6]. Therefore, identifying individuals at high risk of falls in hospitals to take preventive measures is particularly important, especially among older adults.

The MFS (Morse Fall Scale) and STRATIFY (St. Thomas’s Risk Assessment Tool in Falling Elderly Inpatients), widely used in hospitals to identify individuals at high risk of falls, have drawbacks such as low specificity [10,11]. Several studies have developed predictive models for fall risk in older inpatients [12-17]. While some employed traditional regression methods [12-15,17], these conventional approaches often struggle with complex, multidimensional data [18]. Other models exhibit limited applicability, being restricted to specific clinical settings or units [12,13,17]. Additionally, certain models rely solely on clinical texts for prediction, a methodology constrained by single-variable limitations that compromise performance [14].

In recent years, machine learning (ML) algorithms have attracted considerable interest in health care predictive modeling due to their capacity to develop highly accurate prediction models at low cost [19]. The capacity of ML algorithms to process high-dimensional data not only enhances the accuracy and efficiency of predictive models but also enables personalized risk prediction [4,20]. Although existing studies have employed ML algorithms to develop fall prediction models for hospitalized older adults, these models exhibit limitations, including suboptimal performance, applicability restricted to specific geriatric subpopulations, and reliance on environmental detection systems that hinder their widespread clinical adoption [16,21-23]. Critically, limited studies have offered comprehensive explanations or analyses of model predictions, restricting clinical applicability and diminishing the practical value of these models.

Therefore, the objective of our study is to develop and validate multiple ML models utilizing clinically accessible data to predict fall risk of hospitalized older adults. We seek to identify the optimal model while interpreting its predictions through the Shapley Additive Explanations (SHAP) method.

## Methods

### Data Source and Participants

Using an adverse event reporting system integrated into electronic nursing workstations of a tertiary general hospital, researchers retrieved fall incident records for hospitalized older adults (aged ≥60 y) occurring between January 2018 and December 2024, extracting hospitalization identifiers and fall timestamps. An electronic health record (EHR) system was used to record admission and discharge dates along with hospitalization identifiers for older adults without a history of falls hospitalized between January 2018 and December 2024. The fall timestamp of each case patient was used to anchor the index time for the matched controls. For each case, 2 controls were matched. Specifically, we first preprocessed the data by removing duplicate records from individuals with multiple hospitalizations (retaining only the first admission). From this refined pool of potential controls, we then used a Visual Basic algorithm in Microsoft Excel (version 16.0) to identify patients whose entire hospitalization period (from admission to discharge) encompassed the fall timestamp. This approach ensures that both cases and controls were exposed to similar time-dependent clinical factors at the same specific time point, thereby minimizing potential time-dependent bias. The case and control groups were not matched on demographics such as age or gender in order to maintain the natural distribution found in real-world clinical settings. With the aim of capturing all relevant information, variables with clinical or predictive relevance were included as model features for the ML algorithm to parse their associations with the outcome.

Matched controls identified as day cases were excluded and replaced until a 1:2 case-control ratio was maintained. This ratio was selected based on considerations of statistical power, cost-effectiveness, and practical constraints, as increasing the control-to-case ratio beyond 2:1 yields diminishing returns in power while substantially increasing costs and workload [24,25]. Cases were initially identified from the adverse event reporting system as any patient with a documented fall event occurring within the hospital premises and were excluded if they were aged <60 years old at admission, experienced subsequent falls occurring during the same hospitalization, or were nonhospitalized patients or day cases. Controls were selected from the EHR system as hospitalized patients aged ≥60 years with no record of an in-hospital fall and were excluded for having duplicate admission records (only the first was retained), day-case status, or if they could not be matched to a case. We excluded day case patients because more than 20% of the data were missing in EHR. The sample size was estimated using the “pmsampsize” package in R software (version 4.5.0). According to other researchers, the c-statistic is 0.73, the number of predictor parameters chosen for our study is 17, and the prevalence is 0.33 (1/3), with a required sample size of 992 for the calculation.

### Ethical Considerations

The study was approved by the Ethics Committee Board of the Second Affiliated Hospital of Anhui Medical University (approval number: YX2025-162). This study adheres strictly to privacy protection principles. Nonessential identifying information is omitted during data processing, and informed consent is obtained when necessary. Informed consent was waived for patients who died or were disconnected. No financial compensation is provided to participants. This study conforms to the principles outlined in the TRIPOD (Transparent Reporting of a multivariable prediction model for Individual Prognosis or Diagnosis) statement.

### Data Collection and Processing

All records containing timestamps and hospitalization identifiers were randomly split into 2 datasets. Two uniformly trained data collectors independently extracted variables through the EHR system using these identifiers, followed by cross-verification upon completion. Five categories of variables were collected: demographic characteristics, comorbidities, medications, laboratory indicators, and other variables. Table S1 in Multimedia Appendix 1 provides the list of 64 extracted variables. Demographic characteristics and sleep duration data were extracted from hospital admission records. The absence of BMI values was directly attributable to practical barriers in anthropometric data collection for patients with mobility limitations (bedridden or wheelchair-dependent status). Comorbidities were identified by integrating inpatient diagnoses from admission summaries with discharge diagnoses in corresponding discharge records. Medication administration records were retrieved from both permanent and temporary medical orders to capture all medications administered within the 24-hour period preceding the timestamp. Polypharmacy was defined as taking 5 or more medications daily. Laboratory indicators were collected from laboratory test reports. For indicators with repeated measurements, data within the 7 days before and after the timestamp were selected for analysis. The remaining variables were extracted from nursing records within 1 week before and after the timestamp. Given that at least 2 nursing records are documented weekly, there is no missing data for these variables. A total of 64 variables were initially extracted. With 27.49% missing values, BMI was removed from analysis. For the remaining variables, only albumin and hemoglobin contained missing values (0.03% and 0.04%, respectively). The missing values for albumin and hemoglobin were imputed using the random forest (RF) imputation method, implemented via the “missForest” package in R software (version 4.5.0). This approach offers the advantage of handling mixed data types (continuous and categorical) and effectively capturing nonlinear relationships among variables [26].

### Feature Selection

The dataset was randomly split into a training set (70%) and a testing set (30%). A three-step selection strategy was implemented in the training set to identify optimal predictors. First, univariate (LR) was applied for preliminary screening (*P*<.05) to retain statistically significant variables. Second, 5 feature selection methods were integrated: stepwise regression (SR) comprises 3 variants—forward selection, backward selection, and bidirectional elimination; least absolute shrinkage and selection operator (LASSO); and random forest-recursive feature elimination (RF-RFE). Predictors were determined by the overlap among the results of these methods. This approach aimed to mitigate high correlation among predictors while capturing their complex relationships with the outcome variable [27]. SR iteratively adjusts variables based on statistical significance, LASSO addresses high dimensionality and multicollinearity while preventing overfitting, and RF-RFE captures nonlinear patterns and variable interactions. Both LASSO and RF-RFE incorporated 10-fold cross-validation. Finally, clinical experts validated the selected predictors to ensure clinical applicability.

### Models Development and Validation

To comprehensively evaluate predictive performance and ensure robust results, we employed multiple algorithms to construct predictive models in the training set, including seven ML models: LR, support vector machines, RF, gradient boosting machine (GBM), extreme gradient boosting (XGBoost), k-nearest neighbor (KNN), and neural network (NN). Grounded in distinct modeling philosophies, each algorithm offers unique advantages. LR establishes an optimal linear decision boundary, valued for its conceptual simplicity and high interpretability, serving as a reliable performance benchmark [20]. Support vector machines aim to determine a separating hyperplane that maximizes the geometric margin for robust classification. They address nonlinear problems by employing kernel functions to project data into a higher-dimensional feature space where the maximum-margin principle is applied [20]. As a representative bagging ensemble, RF enhances predictive stability and captures complex feature interactions by aggregating numerous decorrelated decision trees, also providing inherent resistance to overfitting and enabling feature importance evaluation [20,28]. GBM employs a sequential modeling strategy that iteratively corrects errors from preceding models, often achieving high predictive accuracy [20]. XGBoost, an optimized implementation of gradient boosting, incorporates regularization and advanced algorithmic techniques to further improve computational efficiency and performance [20]. KNN is an instance-based learning method operating on the principle of local similarity. Predictions are derived from the majority label or average value of a sample’s KNNs in the feature space, offering an intuitive perspective on the local data structure [29]. NN, or deep learning models, function as universal approximators by leveraging multiple layers of tunable nonlinear transformations. This architecture enables them to automatically learn hierarchical data representations and extract complex, high-level features through training [30]. This systematic selection of algorithms, encompassing linear models, kernel methods, bagging and boosting ensembles, instance-based learning, and NNs, ensures our evaluation is comprehensive and avoids bias toward any single modeling strategy.

To mitigate class imbalance, we applied random upsampling to the training dataset, which involves duplicating instances from the minority class at random to balance the class distribution. Subsequently, to rigorously tune hyperparameters and guard against overfitting, we performed a grid search with 10-fold cross-validation on this processed training set to identify the optimal parameters. The test set was used to evaluate model performance. The area under the receiver operating characteristic curve (AUROC) in the testing set served as the primary metric for assessing discriminative ability. Model discrimination was primarily assessed using the AUROC. This metric is considered a standard method for evaluating ranking ability, as it provides a threshold-independent assessment of a model’s inherent discriminative power [31]. Additionally, model performance was comprehensively evaluated using the area under the precision-recall curve (AUPRC), which is particularly informative for imbalanced datasets, along with sensitivity, specificity, accuracy, recall, *F*_1_-score, positive predictive value, and negative predictive value. Calibration curves were plotted to assess prediction accuracy. Decision curve analysis (DCA) was performed to quantify clinical utility. SHAP is a model interpretation tool that calculates feature contribution values to provide both global (model-level) and local (individual prediction) explanations, making models more interpretable and applicable [20,32].

Therefore, we employed the SHAP method to elucidate how individual features influence fall risk predictions in hospitalized older adults within the optimal model.

### Statistical Analysis

All statistical analyses were performed using R software (version 4.5.0), with categories merged when necessary to address sparse data. Use of walking assistance (UWA) was classified into 4 groups: no assistance, wheelchair or bedridden, support by others or furniture, and walker/crutches/cane. Continuous variables were categorized as follows: age into 60 to 69, 70 to 79, and ≥80 years; serum albumin into ≥34 and <34 g/L [33]; MFS scores into <45 points and ≥45 points [34]; modified Barthel Index (mBI) [35] scores into 0 to 20 points, 21 to 60 points, 61 to 90 points, 91 to 99 points, and 100 points; Nutritional Risk Screening 2002 (NRS 2002) scores into <3 points (no nutritional risk) and ≥3 points (at risk) [36]; and Numeric Pain Rating Scale scores into 0 points (no pain) and ≥1 point (pain) [37]. Continuous variables, none of which followed a normal distribution, were expressed as medians and IQR (M, Q1-Q3). Categorical variables were reported as numbers and percentages (n, %). Differences between groups were analyzed using the Mann-Whitney *U* test for nonnormally distributed continuous variables and the Chi-square test (or Fisher exact test for sparse data) for categorical variables. A 2-sided *P*<.05 was considered statistically significant.

## Results

### Baseline Characteristics

**Figure 1.**
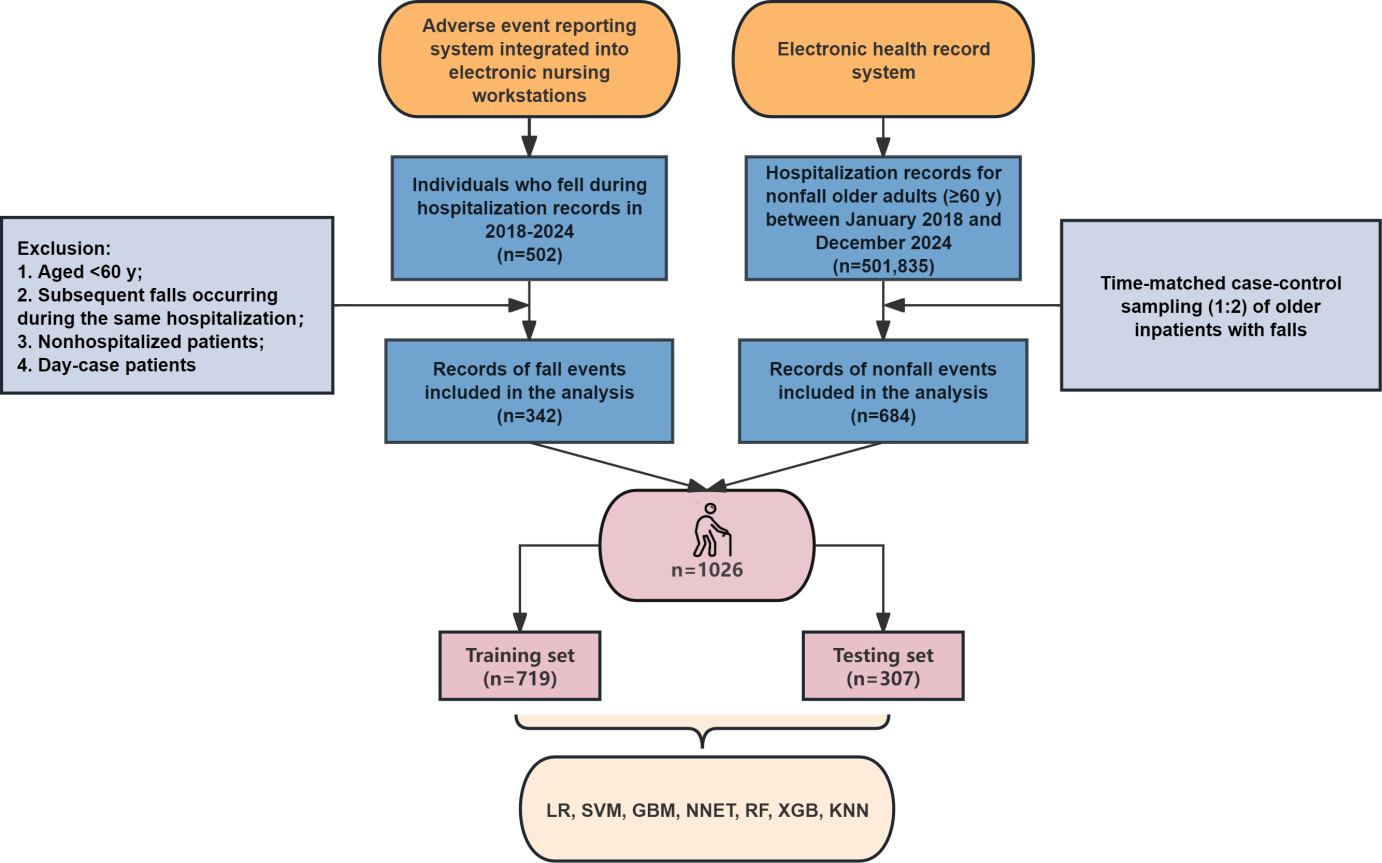
Flowchart of the patient screening. GBM: gradient boosting machine; KNN: k-nearest neighbor; LR: logistic regression; NNET: neural network; RF: random forest; SVM: support vector machine; XGB: extreme gradient boosting.

Ultimately, 1026 older adults were included in the study. Figure 1 illustrates the process of patient screening. The comparison between fallers and nonfallers in the overall dataset is presented in Table S2 in Multimedia Appendix 1. Among the 1026 patients, 40.84% (419/1026) were aged 60 to 69 years and 55.65% (571/1026) were male. Among the 342 fallers, 40.06% (137/342) were aged 70 to 79 years and 52.05% (178/342) were male. Significant differences were observed between fallers and nonfallers in the following variables: age, blood pressure, diabetes, chronic kidney disease, heart failure, hypothyroidism, cancer, Parkinson’s disease, dizziness, stroke, gait abnormality, epilepsy, visual impairment, hearing impairment, polypharmacy, antiplatelet drugs, statins, α-blockers, vasodilators, antidiabetic drugs, anti-Parkinson’s disease drugs, antiepileptic drugs, benzodiazepines, Z-drugs, albumin levels, fall history in the past 3 months, UWA, emergency admission (EA), MFS scores, NRS 2002 scores, mBI scores, number of indwelling catheters (Indw Cath), and departments.

### Selection of Predictor Variables

**Figure 2.**
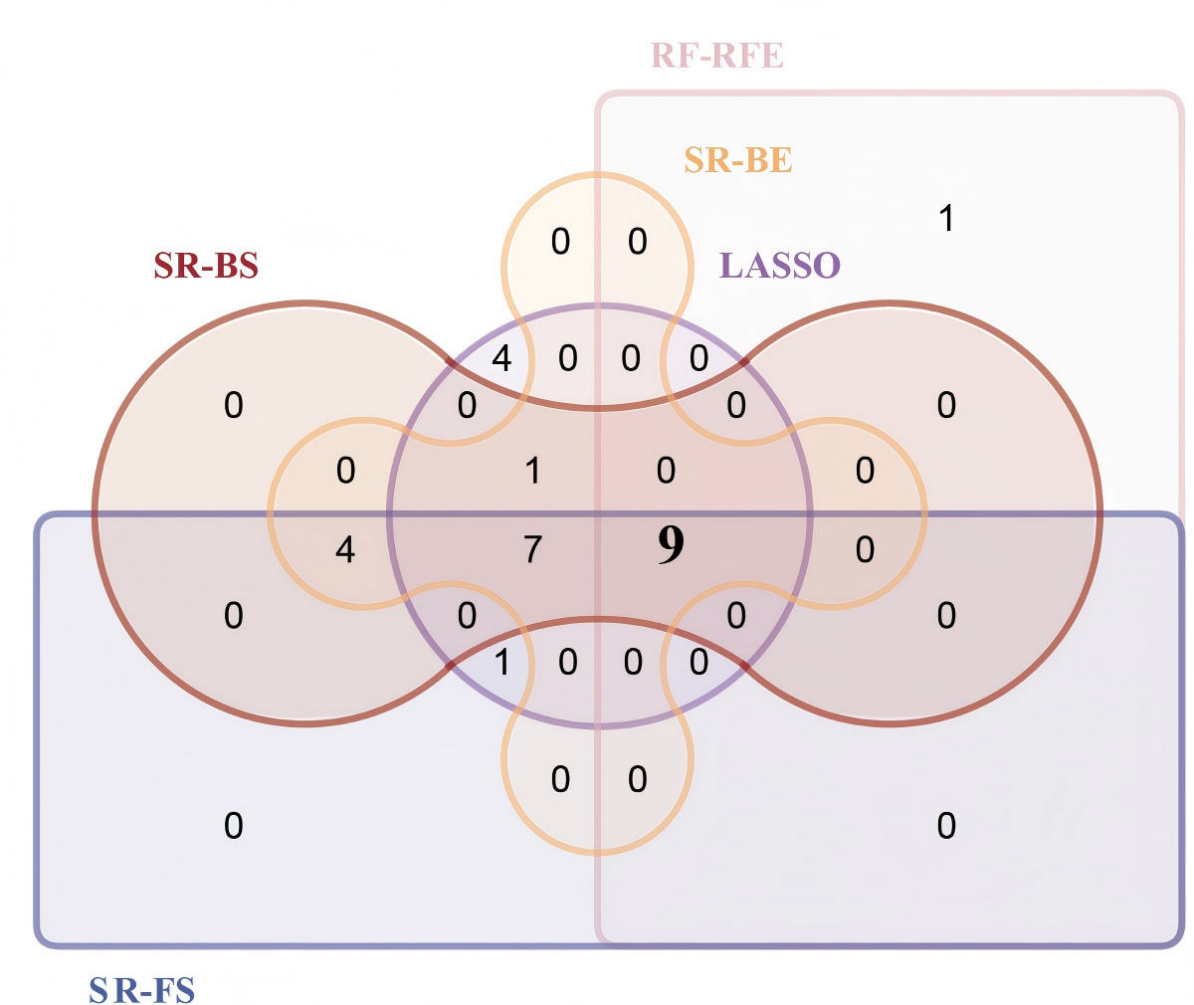
Upset plot of the overlap of predictors selected across 5 methods. BE: bidirectional elimination; BS: backward selection; FS: forward selection, LASSO: least absolute shrinkage and selection operator; RF-RFE: random forest-recursive feature elimination; SR: stepwise regression.

Univariate LR identified 26 potential predictors (*P*<.05) in the training set. The mBI did not reach statistical significance in the univariate analysis, but its *P* value was .054, which is very close to the threshold. Given that the mBI is an important clinical indicator associated with fall risk and to avoid omitting potentially influential factors due to a rigid threshold, we decided after expert discussion to include it as the 27th variable in the subsequent feature selection*,* as detailed in Table S3 of Multimedia Appendix 1. Table 1 displays the predictors identified by the 5 methods (SR-forward selection, SR-backward selection, SR-bidirectional elimination, LASSO, and RF-RFE). Table S4, Figure S1, and Figure S2 in Multimedia Appendix 1 provide detailed information. Figure 2 visualizes the overlap of predictors selected across 5 methods. The intersecting predictors from these methods formed the final predictor set, comprising dizziness, epilepsy, fall history in the past 3 months, UWA, EA, MFS scores, mBI scores, and Indw Cath. After expert consultation, no predictors were added or removed. The final development model included these 8 predictor variables.

**Table 1.** The predictors obtained through 5 selection methods.

Methods	Number of predictors (categories)	Predictor variables
SR-FS	15 (21)	Hypothyroidism, OP^a^, dizziness, stroke, epilepsy, polypharmacy, AC^b^, BZDs^c^, Alb^d^, FH-3M^e^, UWA.1^f^, UWA.2^g^, UWA.3^h^, EA^i^, MFS^j^ scores, mBI.1^k^, mBI.2^l^, mBI.3^m^, mBI.4^n^, Indw Cath.1^o^, Indw Cath.2^p^
SR-BS	15 (21)	Hypothyroidism, OP, dizziness, stroke, epilepsy, AC, BZDs, Zdrugs, Alb, FH-3M, UWA.1, UWA.2, UWA.3, EA, MFS scores, mBI.1, mBI.2, mBI.3, mBI.4, Indw Cath.1, Indw Cath.2
SR-BE	15 (21)	Hypothyroidism, OP, dizziness, stroke, epilepsy, AC, BZDs, Zdrugs, Alb, FH-3M, UWA.1, UWA.2, UWA.3, EA, MFS scores, mBI.1, mBI.2, mBI.3, mBI.4, Indw Cath.1, Indw Cath.2
LASSO	19 (22)	Gender, hypothyroidism, OP, dizziness, stroke, epilepsy, polypharmacy, AC, antidiabetics, BZDs, Zdrugs, Alb, FH-3M, UWA.1, UWA.2, UWA.3, EA, MFS scores, mBI.0^q^, mBI.4, Indw Cath.2, Department.2^r^
RF-RFE	9 (10)	CA^s^, dizziness, epilepsy, FH-3M, UWA.2, UWA.3, EA, MFS scores, mBI.4, Indw Cath.2

a OP: osteoporosis.

b AC: anticoagulants.

c BZDs: benzodiazepines.

d Alb: albumin.

e FH-3M: fall history in the past 3 months.

f UWA.1: use of walking assistance category 1 (wheelchair or bedridden).

g UWA.2: use of walking assistance category 2 (support by others or furniture).

h UWA.3: use of walking assistance category 3 (walker/crutches/cane).

i EA: emergency admission.

j MFS: Morse fall scale.

k mBI.1: modified Barthel Index scores category 1 (21-60 points).

l mBI.2: modified Barthel Index scores category 2 (61-90 points).

m mBI.3: modified Barthel Index scores category 3 (91-99 points).

n mBI.4: modified Barthel Index scores category 4 (100 points).

o Indw Cath.1: number of indwelling catheters 1 (1).

p Indw Cath.2: number of indwelling catheters 2 (≥2).

q mBI.0: modified Barthel Index scores category 0 (0-20 points).

r Department.2: department category 2 (department of rehabilitation medicine).

s CA: cancer.

### Models Development and Validation

**Figure 3.**
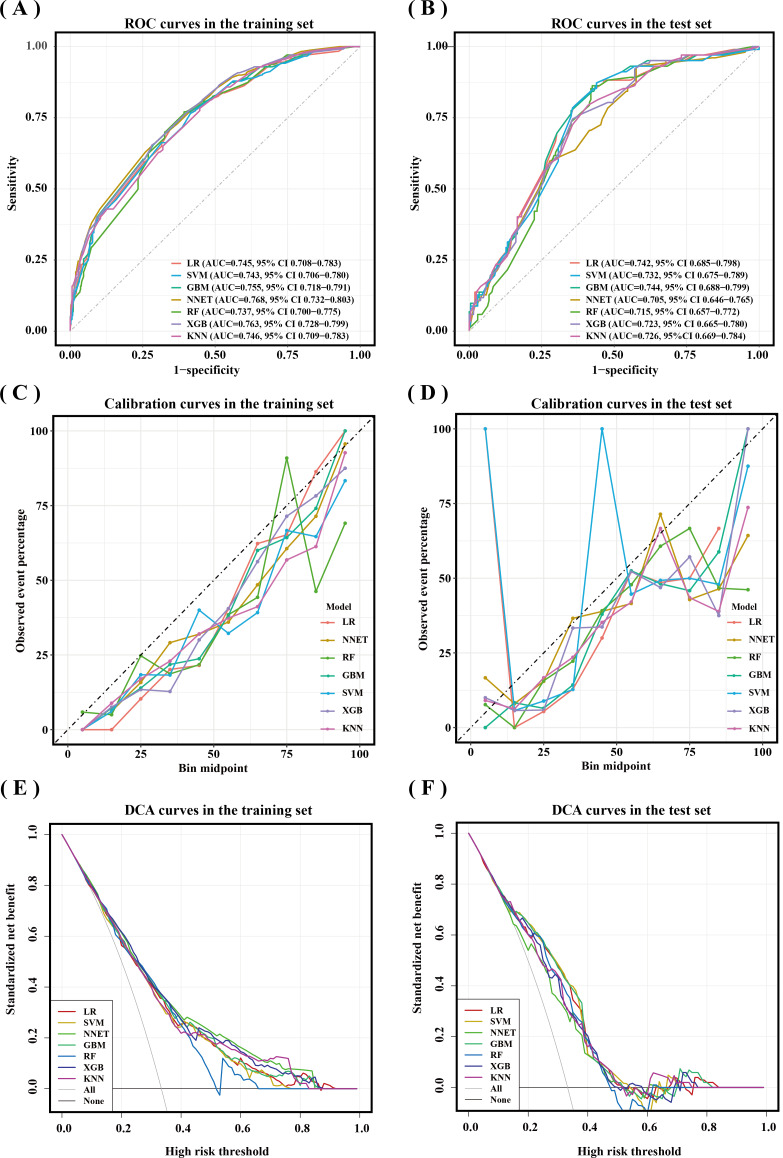
Receiver operating characteristic (ROC) curves, calibration curves, and decision curve analysis (DCA) curves of different machine learning (ML) models in the training and testing sets. (A) ROC curves and area under the ROC curve (AUC) values of different ML prediction models in the training set. (B) ROC curves and AUC values of different ML prediction models in the testing set.(C) Calibration curves of different ML prediction models in the training set. (D) Calibration curves of different ML prediction models in the testing set. (E) DCA curves of different ML prediction models in the training dataset. (F) DCA curves of different ML prediction models in the testing dataset. GBM: gradient boosting machine; KNN: k-nearest neighbor; LR: logistic regression; NNET: neural network; RF: random forest; SVMs: support vector machines; XGBoost: extreme gradient boosting.

Table S5 in Multimedia Appendix 1 compares the characteristics of the training and testing sets. The training set comprised 719 (70%) older adults, while the testing set included 307 (30%). All final model predictors and fall status (yes/no) were balanced between the training and testing sets, as shown in Table 2. The AUROC for the 7 models in the testing set is shown in Figure 3B. Among these, the GBM model demonstrated the highest discrimination with an AUROC of 0.744 (95% CI 0.688‐0.799) compared to the other 6 models. The LR model followed closely with an AUC of 0.742 (95% CI 0.685‐0.798). The NN and RF models had the lowest AUROCs, at 0.705 (95% CI 0.646‐0.765) and 0.715 (95% CI 0.657‐0.772), respectively. Table 3 displays the detailed predictive performance of the 7 ML models. In the testing set, the LR model achieved the highest AUPRC of 0.570 (0.475‐0.663), while the RF model showed the lowest AUPRC of 0.477 (0.386‐0.580). Regarding other performance metrics, the NN models had the best sensitivity (0.931), the XGBoost model had the best specificity (0.644), and the LR model showed the highest accuracy (0.687). The calibration curves for the predictive models in the testing set are shown in Figure 3D. The LR model demonstrated the best calibration ability in the testing set. The DCA curves for the predictive models in the testing set are shown in Figure 3F. The DCA curves suggest that the 7 models have certain clinical utility, generating net benefits within the threshold range of 0 to 0.5. Considering AUROC, sensitivity, and specificity, the GBM was determined to be the best-performing model.

**Table 2.** Characteristics of the predictors in the training and testing sets.

Predictors	Training set (n=719)	Testing set (n=307)	*P* value
Fall, n (%)			>.99
No	479 (66.62)	205 (66.78)	
Yes	240 (33.38)	102 (33.22)	
Dizziness, n (%)			.93
No	705 (98.05)	302 (98.37)	
Yes	14 (1.95)	5 (1.63)	
Epilepsy, n (%)			>.99
No	706 (98.19)	302 (98.37)	
Yes	13 (1.81)	5 (1.63)	
FH-3M^a^, n (%)			.99
No	660 (91.79)	281 (91.53)	
Yes	59 (8.21)	26 (8.47)	
UWA^b^, n (%)			.08
No assistance	475 (66.06)	179 (58.31)	
Wheelchair or bedridden	192 (26.7)	100 (32.57)	
Support by others or furniture	10 (1.39)	3 (0.98)	
Walker/crutches/cane	42 (5.84)	25 (8.14)	
EA^c^, n (%)			.64
No	701 (97.50)	297 (96.74)	
Yes	18 (2.50)	10 (3.26)	
MFS^d^ (points), n (%)			.42
<45	196 (27.26)	92 (29.97)	
≥45	523 (72.74)	215 (70.03)	
mBI^e^ (points), n (%)			.24
0-20	36 (5.01)	13 (4.23)	
21-60	172 (23.92)	93 (30.29)	
61-90	322 (44.78)	121 (39.41)	
91‐99	79 (10.99)	30 (9.77)	
100	110 (15.3)	50 (16.29)	
Indw Cath^f^, n (%)			.81
0	566 (78.72)	247 (80.46)	
1	95 (13.21)	38 (12.38)	
≥2	58 (8.07)	22 (7.17)	

a FH-3M: fall history in the past 3 months.

b UWA: use of walking assistance.

c EA: emergency admission.

d MFS: Morse fall scale.

e mBI: modified Barthel Index.

f Indw Cath: number of indwelling catheters.

**Table 3.** The performance of 7 machine learning models for predicting falls in hospitalized older adults.

Model	AUPRC^a^ (95% CI)	Sensitivity	Specificity	*F*_1_-score	Accuracy	PPV^b^	NPV^c^	Recall
Training set								
LR^d^	0.614 (0.555‐0.672)	0.708	0.656	0.591	0.673	0.508	0.818	0.708
SVMs^e^	0.599 (0.534‐0.661)	0.767	0.585	0.591	0.645	0.480	0.833	0.767
GBM^f^	0.620 (0.559‐0.681)	0.654	0.714	0.588	0.694	0.534	0.805	0.654
NN^g^	0.646 (0.590‐0.705)	0.629	0.741	0.586	0.704	0.549	0.800	0.629
RF^h^	0.580 (0.517‐0.643)	0.771	0.608	0.604	0.662	0.496	0.841	0.771
XGBoost^i^	0.637 (0.579‐0.696)	0.654	0.718	0.590	0.697	0.538	0.806	0.654
KNN^j^	0.626 (0.567‐0.685)	0.783	0.553	0.586	0.630	0.468	0.836	0.783
Testing set								
LR	0.570 (0.475‐0.663)	0.794	0.634	0.628	0.687	0.519	0.861	0.794
SVMs	0.537 (0.437‐0.640)	0.873	0.561	0.634	0.665	0.497	0.898	0.873
GBM	0.560 (0.464‐0.654)	0.873	0.561	0.634	0.665	0.497	0.898	0.873
NN	0.509 (0.409‐0.610)	0.931	0.424	0.603	0.593	0.446	0.926	0.931
RF	0.477 (0.386‐0.580)	0.863	0.576	0.635	0.671	0.503	0.894	0.863
XGBoost	0.535 (0.435‐0.635)	0.745	0.644	0.606	0.678	0.510	0.835	0.745
KNN	0.547 (0.446‐0.643)	0.794	0.590	0.607	0.658	0.491	0.852	0.794

a AUPRC: area under the precision recall curve.

b PPV: positive predictive value.

c NPV: negative predictive value.

d LR: logistic regression.

e SVM: support vector machine.

f GBM: gradient boosting machine.

g NN: neural network.

h RF: random forest.

i XGBoost: extreme gradient boosting.

j KNN: k-nearest neighbor.

### Interpretability Analysis

**Figure 4.**
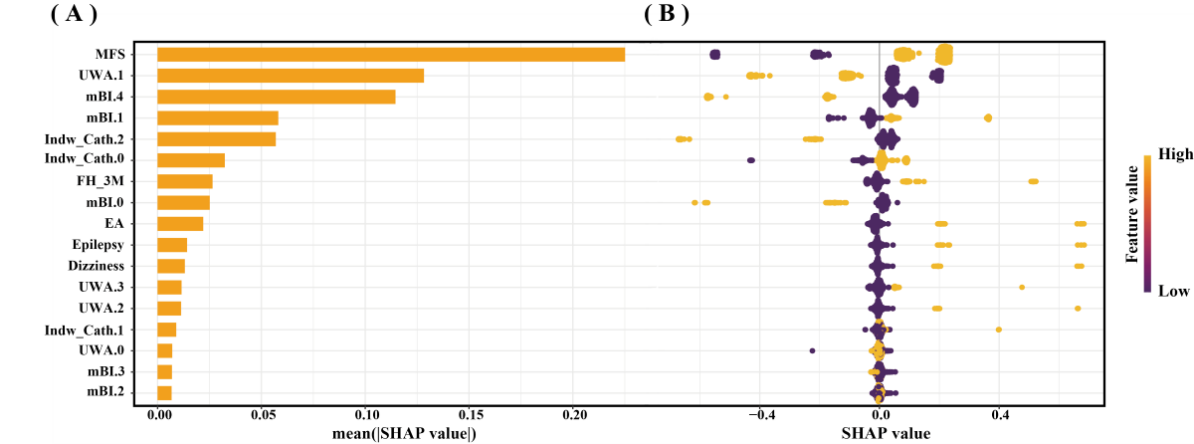
Interpretation of the gradient boosting machine model by the Shapley Additive Explanations (SHAP) method. (A) A bar summary of the most important features according to the SHAP values. (B) Summary and explanation of the most influential features. Yellow dots indicate high-risk values, and purple dots indicate low-risk values. EA: emergency admission; FH-3M: fall history in the past 3 months; Indw Cath.0: number of indwelling catheters 0 (0); Indw Cath.1: number of indwelling catheters 1 (1); Indw Cath.2: number of indwelling catheters 2 (≥2); mBI.0: modified Barthel Index scores category 0 (0-20 points); mBI.1: modified Barthel Index scores category 1 (21-60 points); mBI.2: modified Barthel Index scores category 2 (61‐90 points); mBI.3: modified Barthel Index scores category 3 (91‐99 points); mBI.4: modified Barthel Index scores category 4 (100 points); MFS: Morse Fall Scale; UWA.0: use of walking assistance category 0 (no assistance); UWA.1: use of walking assistance category 1 (wheelchair or bedridden); UWA.2: use of walking assistance category 2 (support by others or furniture); UWA.3: use of walking assistance category 3 (walker/crutches/cane).

**Figure 5.**
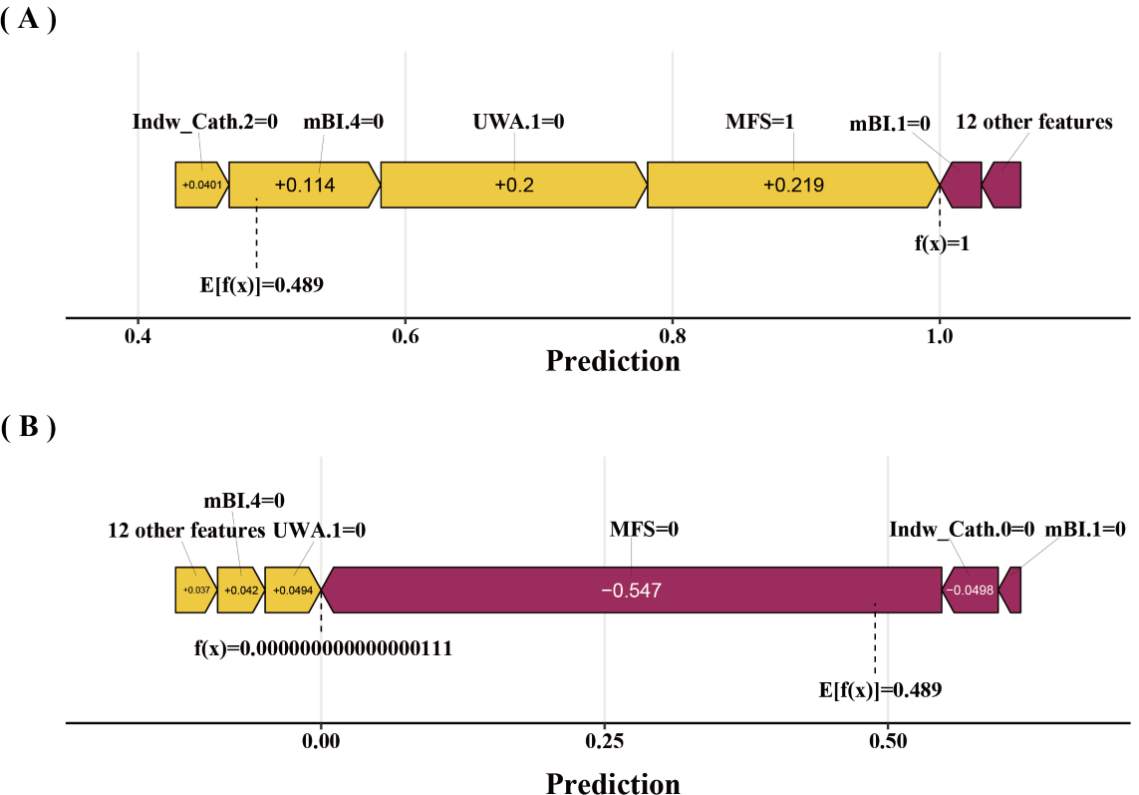
Compositional risk of feature contributions for predicting the occurrence of falls in 2 older adults during hospitalization. Purple arrows denote factors that decrease the risk of falls for old adults during hospitalization, while yellow arrows indicate features that increase the risk. (A) An older adult with fewer than 2 indwelling catheters, a modified Barthel Index (mBI) score not equal to 100 and not within the range of 21‐60 points, not using a wheelchair or bedridden, and a Morse Fall Scale (MFS) score ≥45 points experienced a fall during hospitalization. (B) An older adult with an mBI score not equal to 100 or within the range of 21‐60 points, not using a wheelchair or bedridden, with the presence of indwelling catheters, and an MFS score <45 points did not experience a fall during hospitalization. Indw Cath.0: number of indwelling catheters 0 (0); Indw cath.2: number of indwelling catheters 2 (≥2); mBI.1: modified Barthel Index scores category 1 (21‐60 points); mBI.4: modified Barthel Index scores category 4 (100 points); UWA.1: use of walking assistance category 1 (wheelchair or bedridden).

SHAP was utilized to illustrate how the features predict the occurrence of falls in old adults during hospitalization within the GBM model. Figure 4A displays the 17 features sorted by their average absolute SHAP values, and higher absolute SHAP indicates greater contribution to fall risk. Figure 4B shows the impact values and explanations of these features, and yellow dots represent high risk, while purple dots indicate low risk. An MFS score of ≥45, an mBI score that is not 100 points, an mBI score not between 0 and 20 points, having fewer than 2 indwelling tubes, a history of falls in the past 3 months, EA, epilepsy, dizziness, use of a walker/cane/crutch, requiring assistance from others/furniture for walking, and not using a wheelchair or not being bedridden are associated with a higher risk of falls in old adults during hospitalization. Beyond global SHAP interpretations, local interpretability was demonstrated. Figure 5A and B visualizes how the GBA model makes predictions about falls in older adults during hospitalization; yellow arrows indicate risk-increasing features and purple arrows risk-decreasing features. The *f(x*) values inside arrows quantify each feature’s contribution. Summing these yields the model’s final prediction, which is represented by the *f(x*) value outside arrows.

## Discussion

### Principal Results

In this study, we used 7 ML algorithms to predict in-hospital falls among hospitalized older adults based on clinically accessible data, including demographic characteristics, comorbidities, laboratory parameters, and medications. The GBM algorithm demonstrated the optimal predictive performance. Model interpretability was achieved at both global and individualized patient levels using SHAP [38]. The SHAP approach bridges the gap between ML models and realistic clinical decision-making, enabling health care providers to understand the model’s predictive process and trust its predictive power [39].

In our study, multiple ML algorithms based on distinct principles were employed to develop predictive models. However, the predictive performance across these methods showed limited variation, consistent with prior research [20], which suggests that advanced ML algorithms generally perform well on relatively small and low-dimensional datasets. Through a comprehensive evaluation of the AUROC, AUPRC, sensitivity, and specificity, the GBM model was ultimately selected as the optimal model. Notably, LR also demonstrated competitive performance, and DCA curves indicated that the LR model could provide favorable clinical net benefit. Nevertheless, compared to LR, GBM offers distinct advantages in handling nonlinear relationships and complex data patterns [27].

Fall risk factors among hospitalized older adults encompass multiple domains, including demographic characteristics, comorbidities, laboratory parameters, and medications [40-42]. Previous studies have relied on subjective nursing documentation texts, comprehensive geriatric assessments, or environmental detection systems to develop fall prediction models for hospitalized individuals [16,21], which limits their clinical utility. Identifying predictors is a critical step in building predictive models. It is notable that the predictors identified in our study are aligned with routinely collected clinical data, ensuring practical accessibility in health care settings. Conventional approaches often select predictors using a single method, such as regression models, whereas combining multiple feature selection techniques may yield simplified models with higher generalizability [43]. Different from previous studies, we used multiple methods such as SR, RF-RFE, and LASSO to identify predictors, which is one of the advantages of our study.

Eight variables were ultimately identified: dizziness, epilepsy, fall history within the past 3 months, UWA, EA, MFS scores, mBI scores, and the number of Indw Cath. They are also key predictors in other predictive models [11,15,16,44]. Our study identified MFS scores ≥45, nonbedridden and not using a wheelchair, and scores of mBI not 100 as the 3 strongest predictors of falls in hospitalized older adults. These findings align with previous studies [11,15,16]. MFS is widely used for fall risk assessment in hospitals. Previous research has shown that MFS exhibits lower sensitivity than ML models [11,44]. Nevertheless, including it as a predictor in ML models permits the evaluation of its predictive contribution relative to other variables. MFS remains a valid predictor of falls among hospitalized patients [11]. Similarly, in our study, MFS emerged as a strong feature in the ML model.

Moreover, since patients’ clinical data often include MFS scores, an integrated model that incorporates MFS can better simulate real-world decision-making, providing a more practical foundation for clinical decision. One of the key strengths of our study lies in integrating a simple, widely used assessment tool with a high-performance ML method, leveraging the advantages of both methodologies to develop and validate a simple, easily generalizable predictive model. This study found that older adults who are not bedridden or not using a wheelchair had a higher fall risk during hospitalization. This may occur as over half of falls happen during daily activities [45], whereas bedridden or wheelchair-bound patients have very low activities of daily living (ADL) ability, limiting activity engagement and thereby reducing fall risk. Similarly, patients with mBI scores >0 had higher fall risk, where higher scores indicate better ADL ability [35]. Notably, those with mBI scores <100 or 21 to 60 also showed increased risk, implying a nonlinear relationship between mBI scores and fall risk. This contradicts findings by Dormosh et al [15] and Chu et al [16] that low ADL ability predicts falls but aligns with Nagarkar et al’s [45] longitudinal study linking difficulty with >4 ADL to higher fall odds. Functional decline impairs musculoskeletal integrity and body composition, reducing mobility and increasing fall risk [45-47]. However, the relationship between functional ability and fall risk in elderly patients requires further investigation. Identifying functional states associated with the highest fall risk and implementing dynamic interventions are crucial for preventing falls in this population.

Despite the growing number of ML-based clinical prediction models being developed, most studies lack interpretability of these models, limiting their clinical understanding and practical adoption. The interpretability of ML predictions requires attention from researchers so that physicians can understand, trust, and ultimately apply these predictive models to guide their clinical practice [28,38,39]. SHAP is a model-agnostic interpretation framework grounded in cooperative game theory. Its core lies in computing Shapley values to quantify the marginal contribution of each input feature to individual predictions. This approach provides consistent and locally accurate explanations for every prediction made by the model [38]. In this study, we addressed the “black-box” nature of ML models by implementing SHAP to interpret the GBM model at both global and individualized levels. This means that in a clinical setting, the model can calculate a patient’s fall risk in real time and simultaneously provide the primary clinically interpretable factors contributing to that risk, thereby enabling rapid screening and informed decision-making. SHAP improves the clinical utility of prediction models, providing fall risk prediction and interpretable descriptions for older adults during hospitalization, thereby demonstrating its potential to address the “black-box” problem [28,39].

### Limitations

This study has several limitations. First, the predictive model was developed using single-center retrospective data, which may introduce potential biases and limit its generalizability to other health care settings. External validation in multicenter cohorts is required to confirm broader applicability. Second, incorporating environmental variables (eg, ward layout, lighting conditions) was challenging due to constraints in single center data collection. Lastly, the exclusion of additional laboratory parameters may have overlooked potential predictors. Future research should prioritize integrating environmental variables, expanding laboratory indicators, and leveraging multicenter datasets for model development and validation.

### Conclusions

In this study, multiple ML models were developed and validated using multifaceted clinical data to identify the risk of falls among hospitalized older adults. The GBM model demonstrated the optimal predictive performance. By SHAP, the clinical utility of the predictive model was significantly enhanced. In the future, this GBM fall prediction model could be integrated into the hospital EHR system as an embedded decision support module to dynamically assess fall risk among inpatients and generate real-time alerts. Simultaneously, based on the SHAP values provided by the model, the system could offer evidence to support health care providers in developing personalized intervention measures, thereby translating risk prediction into clinical actions aimed at reducing the incidence of falls in hospitalized older adults.

## Supplementary material

10.2196/80602 Multimedia Appendix 1 The variable list, patient characteristics, regression analysis results, and model development process.

## Abbreviations

ADLs: activities of daily living
AUPRC: area under the precision-recall curve
AUROC: area under the receiver operating characteristic curve
DCA: decision curve analysis
EA: emergency admission
EHR: electronic health record
FH-3M: fall history in the past 3-months
GBM: gradient boosting machine
Indw Cath: indwelling catheters
KNN: k-nearest neighbor
LASSO: least absolute shrinkage and selection operator
LR: logistic regression
mBI: modified Barthel Index
MFS: Morse Fall Scale
ML: machine learning
NN: neural network
NRS 2002: Nutritional Risk Screening 2002
RF: random forest
RF-RFE: random forest-recursive feature elimination
SHAP: Shapley Additive Explanations
SR: stepwise regression
STRATIFY: St. Thomas’s risk assessment tool in falling elderly inpatients
TRIPOD: Transparent Reporting of a multivariable prediction model for Individual Prognosis or Diagnosis
UWA: use of walking assistance
XGBoost: extreme gradient boosting
