# Correction: Development and Validation of Machine Learning Models for Predicting Falls Among Hospitalized Older Adults: Retrospective Cross-Sectional Study

**DOI:** 10.2196/101284

**Published:** 2026-08-13

**Authors:** Xiyao Yang, Juan Ren, Dan Su, Manzhen Bao, Miao Zhang, Xiaoming Chen, Yanhua Li, Zonggui Wang, Xiujing Dai, Zengzeng Wei, Shuiyu Zhang, Yuxin Zhang, Juan Li, Xiaolin Li, Junjin Xu, Nan Mo

**Affiliations:** 1 Second Hospital of Anhui Medical University, No.678 Furong Road, Shushan District, Hefei, 230601, China, 86 1-809-650-2125; 2 School of Nursing, Anhui Medical University, Hefei, China; 3 The Taikang Health and Wellness Industry Research Institute, Anhui Medical University, Hefei, China

In “Development and Validation of Machine Learning Models for Predicting Falls Among Hospitalized Older Adults: Retrospective Cross-Sectional Study” [1], the authors noted one error.

In the Selection of Predictor Variables section, the following sentence was replaced:

*Univariate LR identified 27 potential predictors (P<.05) in the training set, as detailed in Table S3 of Multimedia Appendix 1.*

The sentence now reads:

*Univariate LR identified 26 potential predictors (P<.05) in the training set. The mBI did not reach statistical significance in the univariate analysis, but its P value was .054, which is very close to the threshold. Given that the mBI is an important clinical indicator associated with fall risk and to avoid omitting potentially influential factors due to a rigid threshold, we decided after expert discussion to include it as the 27th variable in the subsequent feature selection, as detailed in Table S3 of Multimedia Appendix 1.*

The correction will appear in the online version of the paper on the JMIR Publications website, together with the publication of this correction notice. Because this was made after submission to PubMed, PubMed Central, and other full-text repositories, the corrected article has also been resubmitted to those repositories.
