# Supplementary material for: Development and Validation of Machine Learning Models for Predicting Falls Among Hospitalized Older Adults: Retrospective Cross-Sectional Study
Source: JMIR Aging. 2026 Jan 5;9:e80602. doi: 10.2196/80602 (PMC12767673; doi:10.2196/80602)
Supplement: Multimedia Appendix 1 [file aging-v9-e80602-s001.docx]

**Multimedia Appendix List**

**Table S1. List of variables and data types.**

**Table S2. Clinical features of patients in the Fall and Non-fall groups.**

**Table S3. Univariate logistic regression in the training set.**

**Table S4. Multivariate stepwise regression analysis of variables.**

**Table S5. Clinical features of patients in the training and testing set.**

**Figure S1. LASSO regression analysis plot.**

**Figure S2. RF-REF model performance varies with feature subset size.**

**Supplementary Table S1**

**Table S1. List of variables and data types.**

| **Number** | **Variable name** | **Data type** | **Number** | **Variable name** | **Data type** |
| --- | --- | --- | --- | --- | --- |
| **Demographic characteristics** | | | 33 | Diuretics | Categorical data |
| 1 | Age | Categorical data | 34 | NSAIDs | Categorical data |
| 2 | Gender | Categorical data | 35 | MMR | Categorical data |
| 3 | Marital status | Categorical data | 36 | Statins | Categorical data |
| 4 | Education | Categorical data | 37 | α-blockers | Categorical data |
| 5 | BMI | Categorical data | 38 | β-blockers | Categorical data |
| **Comorbidities** | | | 39 | Vasodilators | Categorical data |
| 6 | Bp | Categorical data | 40 | Cardiac glycosides | Categorical data |
| 7 | Diabetes | Categorical data | 41 | Antidiabetics | Categorical data |
| 8 | CHD | Categorical data | 42 | Anti-PD | Categorical data |
| 9 | COPD | Categorical data | 43 | Anxiolytics | Categorical data |
| 10 | CKD | Categorical data | 44 | Anticholinergics | Categorical data |
| 11 | HF | Categorical data | 45 | AD (SNRIs/SSRIs/  Tricyclic antidepressants) | Categorical data |
| 12 | Dyslipidemia | Categorical data | 46 | AEDs | Categorical data |
| 13 | Hypothyroidism | Categorical data | 47 | Antipsychotics | Categorical data |
| 14 | Arrhythmia | Categorical data | 48 | BZDs | Categorical data |
| 15 | CA | Categorical data | 49 | Z-drugs | Categorical data |
| 16 | CI | Categorical data | 50 | Opioids | Categorical data |
| 17 | PD | Categorical data | **Laboratory indicators** | | |
| 18 | DSD | Categorical data | 51 | Alb | Categorical data |
| 19 | OP | Categorical data | 52 | Hb | Continuous |
| 20 | Dizziness | Categorical data | **Other variables** | | |
| 21 | Hypotension | Categorical data | 53 | Sleep duration | Continuous |
| 22 | Stroke | Categorical data | 54 | Consciousness | Categorical data |
| 23 | RD | Categorical data | 55 | FH-3M | Categorical data |
| 24 | Epilepsy | Categorical data | 56 | UWA | Categorical data |
| 25 | GA | Categorical data | 57 | EA | Categorical data |
| 26 | VI | Categorical data | 58 | Chaperone | Categorical data |
| 27 | HI | Categorical data | 59 | MFS scores | Categorical data |
| 28 | Sleep disorder | Categorical data | 60 | NRS scores | Categorical data |
| **Medications** | | | 61 | NRS 2002 scores | Categorical data |
| 29 | Polypharmacy | Categorical data | 62 | mBI scores | Categorical data |
| 30 | AC | Categorical data | 63 | Indw Cath | Categorical data |
| 31 | AP | Categorical data | 64 | Department | Categorical data |
| 32 | PPI | Categorical data |  |  |  |

BMI: body mass index; Bp: blood pressure; CHD: coronary heart disease; COPD: chronic obstructive pulmonary disease; CKD: chronic kidney disease; HF: heart failure; CA: cancer; CI: cognitive impairment; PD: Parkinson's disease; DSD: degenerative spinal disease; OP: osteoporosis; RD: rheumatic disease; GA: gait abnormality; VI: visual impairment; HI: hearing impairment; AC: anticoagulants; AP: antiplatelet; PPI: proton pump inhibitors; NSAIDs: non-steroidal anti-inflammatory drugs; MMR: muscle relaxants; AD: antidepressants; SNRIs: serotonin and norepinephrine reuptake inhibitors; SSRIs: selective serotonin reuptake inhibitors; AEDs: antiepileptic drugs; BZDs: benzodiazepines; Alb: albumin; Hb: hemoglobin; FH-3M: fall history in the past 3 months; UWA: use of walking assistance; EA: emergency admission; MFS: Morse fall scale; NRS: numeric pain rating scale; NRS 2002: nutritional risk screening 2002; mBI: modified Barthel Index of activities of daily living; Indw Cath: number of indwelling catheters.

**Supplementary Table S2**

**Table S2. Clinical features of patients in the Fall and Non-fall groups.**

| **Variables** | **Total (n = 1026)** | **Non-fall (n = 684)** | **Fall (n = 342)** | ***P*-value** |
| --- | --- | --- | --- | --- |
| **Demographic characteristics** | |  |  |  |
| Age (years), n (%) |  |  |  | < 0.001 |
| 60~69 | 419 (40.84) | 307 (44.88) | 112 (32.75) |  |
| 70~79 | 409 (39.86) | 272 (39.77) | 137 (40.06) |  |
| ≥80 | 198 (19.30) | 105 (15.35) | 93 (27.19) |  |
| Gender, n (%) |  |  |  | 0.115 |
| Male | 571 (55.65) | 393 (57.46) | 178 (52.05) |  |
| Female | 455 (44.35) | 291 (42.54) | 164 (47.95) |  |
| Marital status, n (%) |  |  |  | 0.248 |
| Married | 855 (83.33) | 577 (84.36) | 278 (81.29) |  |
| Non married | 171 (16.67) | 107 (15.64) | 64 (18.71) |  |
| Education, n (%) |  |  |  | 0.322 |
| Primary education or below | 627 (61.11) | 426 (62.28) | 201 (58.77) |  |
| High school or vocational secondary school | 326 (31.77) | 207 (30.26) | 119 (34.80) |  |
| Tertiary education or above | 73 (7.12) | 51 (7.46) | 22 (6.43) |  |
| **Comorbidities** |  |  |  |  |
| Bp, n (%) |  |  |  | 0.036 |
| No | 511 (49.81) | 357 (52.19) | 154 (45.03) |  |
| Yes | 515 (50.19) | 327 (47.81) | 188 (54.97) |  |
| Diabetes, n (%) |  |  |  | < 0.001 |
| No | 760 (74.07) | 529 (77.34) | 231 (67.54) |  |
| Yes | 266 (25.93) | 155 (22.66) | 111 (32.46) |  |
| CHD, n (%) |  |  |  | 0.451 |
| No | 862 (84.02) | 570 (83.33) | 292 (85.38) |  |
| Yes | 164 (15.98) | 114 (16.67) | 50 (14.62) |  |
| COPD, n (%) |  |  |  | 0.078 |
| No | 991 (96.59) | 666 (97.37) | 325 (95.03) |  |
| Yes | 35 (3.41) | 18 (2.63) | 17 (4.97) |  |
| CKD, n (%) |  |  |  | < 0.001 |
| No | 973 (94.83) | 661 (96.64) | 312 (91.23) |  |
| Yes | 53 (5.17) | 23 (3.36) | 30 (8.77) |  |
| HF, n (%) |  |  |  | 0.002 |
| No | 969 (94.44) | 657 (96.05) | 312 (91.23) |  |
| Yes | 57 (5.56) | 27 (3.95) | 30 (8.77) |  |
| Dyslipidemia, n (%) |  |  |  | 0.703 |
| No | 982 (95.71) | 653 (95.47) | 329 (96.20) |  |
| Yes | 44 (4.29) | 31 (4.53) | 13 (3.80) |  |
| Hypothyroidism, n (%) |  |  |  | 0.017 |
| No | 988 (96.30) | 666 (97.37) | 322 (94.15) |  |
| Yes | 38 (3.70) | 18 (2.63) | 20 (5.85) |  |
| Arrhythmia, n (%) |  |  |  | 0.284 |
| No | 845 (82.36) | 570 (83.33) | 275 (80.41) |  |
| Yes | 181 (17.64) | 114 (16.67) | 67 (19.59) |  |
| CA, n (%) |  |  |  | 0.016 |
| No | 660 (64.33) | 422 (61.70) | 238 (69.59) |  |
| Yes | 366 (35.67) | 262 (38.30) | 104 (30.41) |  |
| CI, n (%) |  |  |  | 0.126 |
| No | 1018 (99.22) | 681 (99.56) | 337 (98.54) |  |
| Yes | 8 (0.78) | 3 (0.44) | 5 (1.46) |  |
| PD, n (%) |  |  |  | 0.049 |
| No | 1015 (98.93) | 680 (99.42) | 335 (97.95) |  |
| Yes | 11 (1.07) | 4 (0.58) | 7 (2.05) |  |
| DSD, n (%) |  |  |  | 0.693 |
| No | 985 (96.00) | 655 (95.76) | 330 (96.49) |  |
| Yes | 41 (4.00) | 29 (4.24) | 12 (3.51) |  |
| OP, n (%) |  |  |  | 0.057 |
| No | 1004 (97.86) | 674 (98.54) | 330 (96.49) |  |
| Yes | 22 (2.14) | 10 (1.46) | 12 (3.51) |  |
| Dizziness, n (%) |  |  |  | 0.002 |
| No | 1007 (98.15) | 678 (99.12) | 329 (96.20) |  |
| Yes | 19 (1.85) | 6 (0.88) | 13 (3.80) |  |
| Hypotension, n (%) |  |  |  | 0.099 |
| No | 1020 (99.42) | 682 (99.71) | 338 (98.83) |  |
| Yes | 6 (0.58) | 2 (0.29) | 4 (1.17) |  |
| Stroke, n (%) |  |  |  | < 0.001 |
| No | 745 (72.61) | 521 (76.17) | 224 (65.5) |  |
| Yes | 281 (27.39) | 163 (23.83) | 118 (34.5) |  |
| RD, n (%) |  |  |  | 0.175 |
| No | 956 (93.18) | 643 (94.01) | 313 (91.52) |  |
| Yes | 70 (6.82) | 41 (5.99) | 29 (8.48) |  |
| Epilepsy, n (%) |  |  |  | < 0.001 |
| No | 1008 (98.25) | 680 (99.42) | 328 (95.91) |  |
| Yes | 18 (1.75) | 4 (0.58) | 14 (4.09) |  |
| GA, n (%) |  |  |  | 0.009 |
| No | 958 (93.37) | 649 (94.88) | 309 (90.35) |  |
| Yes | 68 (6.63) | 35 (5.12) | 33 (9.65) |  |
| VI, n (%) |  |  |  | 0.035 |
| No | 457 (44.54) | 321 (46.93) | 136 (39.77) |  |
| Yes | 569 (55.46) | 363 (53.07) | 206 (60.23) |  |
| HI, n (%) |  |  |  | < 0.001 |
| No | 716 (69.79) | 501 (73.25) | 215 (62.87) |  |
| Yes | 310 (30.21) | 183 (26.75) | 127 (37.13) |  |
| Sleep disorder, n (%) |  |  |  | 0.564 |
| No | 819 (79.82) | 550 (80.41) | 269 (78.65) |  |
| Yes | 207 (20.18) | 134 (19.59) | 73 (21.35) |  |
| **Medications** |  |  |  |  |
| Polypharmacy, n (%) |  |  |  | < 0.001 |
| No | 661 (64.42) | 477 (69.74) | 184 (53.80) |  |
| Yes | 365 (35.58) | 207 (30.26) | 158 (46.20) |  |
| AC, n (%) |  |  |  | 0.071 |
| No | 868 (84.60) | 589 (86.11) | 279 (81.58) |  |
| Yes | 158 (15.40) | 95 (13.89) | 63 (18.42) |  |
| AP, n (%) |  |  |  | 0.006 |
| No | 847 (82.55) | 581 (84.94) | 266 (77.78) |  |
| Yes | 179 (17.45) | 103 (15.06) | 76 (22.22) |  |
| PPI, n (%) |  |  |  | 0.192 |
| No | 701 (68.32) | 477 (69.74) | 224 (65.5) |  |
| Yes | 325 (31.68) | 207 (30.26) | 118 (34.5) |  |
| Diuretics, n (%) |  |  |  | 0.104 |
| No | 800 (77.97) | 544 (79.53) | 256 (74.85) |  |
| Yes | 226 (22.03) | 140 (20.47) | 86 (25.15) |  |
| NSAIDs, n (%) |  |  |  | 0.608 |
| No | 965 (94.05) | 641 (93.71) | 324 (94.74) |  |
| Yes | 61 (5.95) | 43 (6.29) | 18 (5.26) |  |
| MMR, n (%) |  |  |  | 0.340 |
| No | 1021 (99.51) | 682 (99.71) | 339 (99.12) |  |
| Yes | 5 (0.49) | 2 (0.29) | 3 (0.88) |  |
| Statins, n (%) |  |  |  | 0.017 |
| No | 802 (78.17) | 550 (80.41) | 252 (73.68) |  |
| Yes | 224 (21.83) | 134 (19.59) | 90 (26.32) |  |
| α blockers, n (%) |  |  |  | 0.026 |
| No | 977 (95.22) | 659 (96.35) | 318 (92.98) |  |
| Yes | 49 (4.78) | 25 (3.65) | 24 (7.02) |  |
| β blockers, n (%) |  |  |  | 0.053 |
| No | 903 (88.01) | 612 (89.47) | 291 (85.09) |  |
| Yes | 123 (11.99) | 72 (10.53) | 51 (14.91) |  |
| Vasodilators, n (%) |  |  |  | 0.047 |
| No | 645 (62.87) | 445 (65.06) | 200 (58.48) |  |
| Yes | 381 (37.13) | 239 (34.94) | 142 (41.52) |  |
| Cardiac glycosides, n (%) |  |  |  | 0.949 |
| No | 995 (96.98) | 664 (97.08) | 331 (96.78) |  |
| Yes | 31 (3.02) | 20 (2.92) | 11 (3.22) |  |
| Antidiabetics, n (%) |  |  |  | < 0.001 |
| No | 822 (80.12) | 574 (83.92) | 248 (72.51) |  |
| Yes | 204 (19.88) | 110 (16.08) | 94 (27.49) |  |
| Anti PD, n (%) |  |  |  | 0.045 |
| No | 1019 (99.32) | 682 (99.71) | 337 (98.54) |  |
| Yes | 7 (0.68) | 2 (0.29) | 5 (1.46) |  |
| Anxiolytics, n (%) |  |  |  | 0.315 |
| No | 1016 (99.03) | 679 (99.27) | 337 (98.54) |  |
| Yes | 10 (0.97) | 5 (0.73) | 5 (1.46) |  |
| Anticholinergics, n (%) |  |  |  | 0.635 |
| No | 998 (97.27) | 667 (97.51) | 331 (96.78) |  |
| Yes | 28 (2.73) | 17 (2.49) | 11 (3.22) |  |
| AD, n (%) |  |  |  | 0.342 |
| No | 1009 (98.34) | 675 (98.68) | 334 (97.66) |  |
| Yes | 17 (1.66) | 9 (1.32) | 8 (2.34) |  |
| AEDs, n (%) |  |  |  | 0.002 |
| No | 988 (96.30) | 668 (97.66) | 320 (93.57) |  |
| Yes | 38 (3.70) | 16 (2.34) | 22 (6.43) |  |
| Antipsychotics, n (%) |  |  |  | 0.783 |
| No | 1011 (98.54) | 673 (98.39) | 338 (98.83) |  |
| Yes | 15 (1.46) | 11 (1.61) | 4 (1.17) |  |
| BZDs, n (%) |  |  |  | 0.019 |
| No | 978 (95.32) | 660 (96.49) | 318 (92.98) |  |
| Yes | 48 (4.68) | 24 (3.51) | 24 (7.02) |  |
| Zdrugs, n (%) |  |  |  | 0.013 |
| No | 1013 (98.73) | 680 (99.42) | 333 (97.37) |  |
| Yes | 13 (1.27) | 4 (0.58) | 9 (2.63) |  |
| Opioids, n (%) |  |  |  | 0.895 |
| No | 957 (93.27) | 637 (93.13) | 320 (93.57) |  |
| Yes | 69 (6.73) | 47 (6.87) | 22 (6.43) |  |
| **Laboratory indicators** |  |  |  |  |
| Alb, n (%) |  |  |  | 0.002 |
| ≥34 | 606 (59.06) | 428 (62.57) | 178 (52.05) |  |
| <34 | 420 (40.94) | 256 (37.43) | 164 (47.95) |  |
| Hb, Median (Q_1_, Q_3_) | 115 (97.27, 127) | 115 (99, 127) | 113.56 (96, 126) | 0.285 |
| **Other variables** |  |  |  |  |
| Sleep duration, Median (Q_1_, Q_3_) | 6.5 (5.5, 7) | 6.5 (5.5, 7) | 6.5 (5.5, 7) | 0.802 |
| Consciousness, n (%) |  |  |  | 0.827 |
| Conscious | 1002 (97.66) | 667 (97.51) | 335 (97.95) |  |
| Unconscious | 24 (2.34) | 17 (2.49) | 7 (2.05) |  |
| FH-3M, n (%) |  |  |  | < 0.001 |
| No | 941 (91.72) | 648 (94.74) | 293 (85.67) |  |
| Yes | 85 (8.28) | 36 (5.26) | 49 (14.33) |  |
| UWA, n (%) |  |  |  | < 0.001 |
| No assistance | 654 (63.74) | 449 (65.64) | 205 (59.94) |  |
| Wheelchair or bedridden | 292 (28.46) | 201 (29.39) | 91 (26.61) |  |
| Support by others or furniture | 13 (1.27) | 5 (0.73) | 8 (2.34) |  |
| Walker/crutches/cane | 67 (6.53) | 29 (4.24) | 38 (11.11) |  |
| EA, n (%) |  |  |  | < 0.001 |
| No | 998 (97.27) | 680 (99.42) | 318 (92.98) |  |
| Yes | 28 (2.73) | 4 (0.58) | 24 (7.02) |  |
| Chaperone, n (%) |  |  |  | 0.286 |
| Unattended | 51 (4.97) | 38 (5.56) | 13 (3.8) |  |
| With caregiver | 975 (95.03) | 646 (94.44) | 329 (96.2) |  |
| MFS (points), n (%) |  |  |  | < 0.001 |
| <45 | 288 (28.07) | 245 (35.82) | 43 (12.57) |  |
| ≥45 | 738 (71.93) | 439 (64.18) | 299 (87.43) |  |
| NRS (points), n (%) |  |  |  | 0.469 |
| 0 | 804 (78.36) | 531 (77.63) | 273 (79.82) |  |
| ≥1 | 222 (21.64) | 153 (22.37) | 69 (20.18) |  |
| NRS 2002 (points), n (%) |  |  |  | 0.009 |
| <3 | 597 (58.19) | 418 (61.11) | 179 (52.34) |  |
| ≥3 | 429 (41.81) | 266 (38.89) | 163 (47.66) |  |
| mBI (points), n (%) |  |  |  | < 0.001 |
| 0~20 | 49 (4.78) | 37 (5.41) | 12 (3.51) |  |
| 21~60 | 265 (25.83) | 162 (23.68) | 103 (30.12) |  |
| 61~90 | 443 (43.18) | 280 (40.94) | 163 (47.66) |  |
| 91-99 | 109 (10.62) | 70 (10.23) | 39 (11.40) |  |
| 100 | 160 (15.59) | 135 (19.74) | 25 (7.31) |  |
| Indw Cath, n (%) |  |  |  | < 0.001 |
| 0 | 813 (79.24) | 524 (76.61) | 289 (84.5) |  |
| 1 | 133 (12.96) | 88 (12.87) | 45 (13.16) |  |
| ≥2 | 80 (7.80) | 72 (10.53) | 8 (2.34) |  |
| Department, n (%) |  |  |  | < 0.001 |
| Department of internal medicine | 639 (62.28) | 407 (59.5) | 232 (67.84) |  |
| Department of surgery | 301 (29.34) | 226 (33.04) | 75 (21.93) |  |
| Department of rehabilitation medicine | 50 (4.87) | 22 (3.22) | 28 (8.19) |  |
| Miscellaneous specialties | 36 (3.51) | 29 (4.24) | 7 (2.05) |  |

Q₁: 1st Quartile; Q₃: 3st Quartile.

**Supplementary Table S3**

**Table S3. Univariate logistic regression in the training set.**

| **Variables** | **Total (n = 719)** | **Non fall (n = 479)** | **Fall (n = 240)** | **OR (95% CI)** | ***P*-value** |
| --- | --- | --- | --- | --- | --- |
| **Demographic characteristics** |  |  |  |  |  |
| Age (years), n (%) |  |  |  |  |  |
| 60~69 | 291 (40.47) | 208 (43.42) | 83 (34.58) | Ref | - |
| 70~79 | 286 (39.78) | 190 (39.67) | 96 (40.00) | 1.27 (0.89, 1.80) | 0.191 |
| ≥80 | 142 (19.75) | 81 (16.91) | 61 (25.42) | 1.89 (1.24, 2.87) | 0.003 |
| Gender, n (%) |  |  |  |  |  |
| Male | 401 (55.77) | 281 (58.66) | 120 (50.00) | Ref | - |
| Female | 318 (44.23) | 198 (41.34) | 120 (50.00) | 1.42 (1.04, 1.94) | 0.028 |
| Marital, n (%) |  |  |  |  |  |
| Married | 603 (83.87) | 405 (84.55) | 198 (82.50) | Ref | - |
| Non married | 116 (16.13) | 74 (15.45) | 42 (17.50) | 1.16 (0.77, 1.76) | 0.481 |
| Education, n (%) |  |  |  |  |  |
| Primary education or below | 436 (60.64) | 297 (62.00) | 139 (57.92) | Ref | - |
| High school or vocational secondary school | 233 (32.41) | 147 (30.69) | 86 (35.83) | 1.25 (0.90, 1.75) | 0.190 |
| Tertiary education or above | 50 (6.95) | 35 (7.31) | 15 (6.25) | 0.92 (0.48, 1.73) | 0.787 |
| **Comorbidities** |  |  |  |  |  |
| Bp, n (%) |  |  |  |  |  |
| No | 354 (49.24) | 247 (51.57) | 107 (44.58) | Ref | - |
| Yes | 365 (50.76) | 232 (48.43) | 133 (55.42) | 1.32 (0.97, 1.81) | 0.078 |
| Diabetes, n (%) |  |  |  |  |  |
| No | 538 (74.83) | 374 (78.08) | 164 (68.33) | Ref | - |
| Yes | 181 (25.17) | 105 (21.92) | 76 (31.67) | 1.65 (1.17, 2.34) | 0.005 |
| CHD, n (%) |  |  |  |  |  |
| No | 603 (83.87) | 397 (82.88) | 206 (85.83) | Ref | - |
| Yes | 116 (16.13) | 82 (17.12) | 34 (14.17) | 0.80 (0.52, 1.23) | 0.311 |
| COPD, n (%) |  |  |  |  |  |
| No | 694 (96.52) | 465 (97.08) | 229 (95.42) | Ref | - |
| Yes | 25 (3.48) | 14 (2.92) | 11 (4.58) | 1.60 (0.71, 3.57) | 0.256 |
| CKD, n (%) |  |  |  |  |  |
| No | 681 (94.71) | 460 (96.03) | 221 (92.08) | Ref | - |
| Yes | 38 (5.29) | 19 (3.97) | 19 (7.92) | 2.08 (1.08, 4.01) | 0.028 |
| HF, n (%) |  |  |  |  |  |
| No | 683 (94.99) | 459 (95.82) | 224 (93.33) | Ref | - |
| Yes | 36 (5.01) | 20 (4.18) | 16 (6.67) | 1.64 (0.83, 3.22) | 0.152 |
| Dyslipidemia, n (%) |  |  |  |  |  |
| No | 694 (96.52) | 460 (96.03) | 234 (97.50) | Ref | - |
| Yes | 25 (3.48) | 19 (3.97) | 6 (2.50) | 0.62 (0.24, 1.58) | 0.316 |
| Hypothyroidism, n (%) |  |  |  |  |  |
| No | 696 (96.80) | 469 (97.91) | 227 (94.58) | Ref | - |
| Yes | 23 (3.20) | 10 (2.09) | 13 (5.42) | 2.69 (1.16, 6.22) | 0.021 |
| Arrhythmia, n (%) |  |  |  |  |  |
| No | 583 (81.08) | 392 (81.84) | 191 (79.58) | Ref | - |
| Yes | 136 (18.92) | 87 (18.16) | 49 (20.42) | 1.16 (0.78, 1.71) | 0.467 |
| CA, n (%) |  |  |  |  |  |
| No | 442 (61.47) | 281 (58.66) | 161 (67.08) | Ref | - |
| Yes | 277 (38.53) | 198 (41.34) | 79 (32.92) | 0.70 (0.50, 0.96) | 0.029 |
| CI, n (%) |  |  |  |  |  |
| No | 712 (99.03) | 477 (99.58) | 235 (97.92) | Ref | - |
| Yes | 7 (0.97) | 2 (0.42) | 5 (2.08) | 5.07 (0.98, 26.35) | 0.053 |
| PD, n (%) |  |  |  |  |  |
| No | 711 (98.89) | 476 (99.37) | 235 (97.92) | Ref | - |
| Yes | 8 (1.11) | 3 (0.63) | 5 (2.08) | 3.38 (0.80, 14.25) | 0.098 |
| DSD, n (%) |  |  |  |  |  |
| No | 692 (96.24) | 462 (96.45) | 230 (95.83) | Ref | - |
| Yes | 27 (3.76) | 17 (3.55) | 10 (4.17) | 1.18 (0.53, 2.62) | 0.681 |
| OP, n (%) |  |  |  |  |  |
| No | 707 (98.33) | 475 (99.16) | 232 (96.67) |  |  |
| Yes | 12 (1.67) | 4 (0.84) | 8 (3.33) | 4.09 (1.22, 13.74) | 0.022 |
| Dizziness, n (%) |  |  |  |  |  |
| No | 705 (98.05) | 476 (99.37) | 229 (95.42) | Ref | - |
| Yes | 14 (1.95) | 3 (0.63) | 11 (4.58) | 7.62 (2.11, 27.59) | 0.002 |
| Hypotension, n (%) |  |  |  |  |  |
| No | 713 (99.17) | 477 (99.58) | 236 (98.33) | Ref | - |
| Yes | 6 (0.83) | 2 (0.42) | 4 (1.67) | 4.04 (0.74, 22.23) | 0.108 |
| Stroke, n (%) |  |  |  |  |  |
| No | 525 (73.02) | 369 (77.04) | 156 (65.00) | Ref | - |
| Yes | 194 (26.98) | 110 (22.96) | 84 (35.00) | 1.81 (1.29, 2.54) | <.001 |
| RD, n (%) |  |  |  |  |  |
| No | 671 (93.32) | 450 (93.95) | 221 (92.08) | Ref | - |
| Yes | 48 (6.68) | 29 (6.05) | 19 (7.92) | 1.33 (0.73, 2.43) | 0.347 |
| Epilepsy, n (%) |  |  |  |  |  |
| No | 706 (98.19) | 476 (99.37) | 230 (95.83) | Ref | - |
| Yes | 13 (1.81) | 3 (0.63) | 10 (4.17) | 6.90 (1.88, 25.31) | 0.004 |
| GA, n (%) |  |  |  |  |  |
| No | 662 (92.07) | 449 (93.74) | 213 (88.75) | Ref | - |
| Yes | 57 (7.93) | 30 (6.26) | 27 (11.25) | 1.90 (1.10, 3.27) | 0.021 |
| VI, n (%) |  |  |  |  |  |
| No | 325 (45.20) | 228 (47.60) | 97 (40.42) | Ref | - |
| Yes | 394 (54.80) | 251 (52.40) | 143 (59.58) | 1.34 (0.98, 1.83) | 0.068 |
| HI, n (%) |  |  |  |  |  |
| No | 503 (69.96) | 346 (72.23) | 157 (65.42) | Ref | - |
| Yes | 216 (30.04) | 133 (27.77) | 83 (34.58) | 1.38 (0.99, 1.92) | 0.060 |
| Sleep disorder, n (%) |  |  |  |  |  |
| No | 585 (81.36) | 394 (82.25) | 191 (79.58) | Ref | - |
| Yes | 134 (18.64) | 85 (17.75) | 49 (20.42) | 1.19 (0.80, 1.76) | 0.386 |
| **Medications** |  |  |  |  |  |
| Polypharmacy, n (%) |  |  |  |  |  |
| No | 461 (64.12) | 331 (69.1) | 130 (54.17) | Ref | - |
| Yes | 258 (35.88) | 148 (30.9) | 110 (45.83) | 1.89 (1.37, 2.60) | <.001 |
| AC, n (%) |  |  |  |  |  |
| No | 617 (85.81) | 423 (88.31) | 194 (80.83) |  |  |
| Yes | 102 (14.19) | 56 (11.69) | 46 (19.17) | 1.79 (1.17, 2.74) | 0.007 |
| AP, n (%) |  |  |  |  |  |
| No | 600 (83.45) | 412 (86.01) | 188 (78.33) | Ref | - |
| Yes | 119 (16.55) | 67 (13.99) | 52 (21.67) | 1.70 (1.14, 2.54) | 0.009 |
| PPI, n (%) |  |  |  |  |  |
| No | 491 (68.29) | 333 (69.52) | 158 (65.83) | Ref | - |
| Yes | 228 (31.71) | 146 (30.48) | 82 (34.17) | 1.18 (0.85, 1.65) | 0.317 |
| Diuretics, n (%) |  |  |  |  |  |
| No | 562 (78.16) | 383 (79.96) | 179 (74.58) | Ref | - |
| Yes | 157 (21.84) | 96 (20.04) | 61 (25.42) | 1.36 (0.94, 1.96) | 0.101 |
| NSAIDs, n (%) |  |  |  |  |  |
| No | 673 (93.60) | 448 (93.53) | 225 (93.75) | Ref | - |
| Yes | 46 (6.40) | 31 (6.47) | 15 (6.25) | 0.96 (0.51, 1.82) | 0.909 |
| MMR, n (%) |  |  |  |  |  |
| No | 714 (99.30) | 477 (99.58) | 237 (98.75) | Ref | - |
| Yes | 5 (0.70) | 2 (0.42) | 3 (1.25) | 3.02 (0.50, 18.19) | 0.228 |
| Statins, n (%) |  |  |  |  |  |
| No | 572 (79.55) | 392 (81.84) | 180 (75.00) | Ref | - |
| Yes | 147 (20.45) | 87 (18.16) | 60 (25.00) | 1.50 (1.03, 2.18) | 0.033 |
| α blockers, n (%) |  |  |  |  |  |
| No | 683 (94.99) | 459 (95.82) | 224 (93.33) | Ref | - |
| Yes | 36 (5.01) | 20 (4.18) | 16 (6.67) | 1.64 (0.83, 3.22) | 0.152 |
| β blockers, n (%) |  |  |  |  |  |
| No | 636 (88.46) | 429 (89.56) | 207 (86.25) | Ref | - |
| Yes | 83 (11.54) | 50 (10.44) | 33 (13.75) | 1.37 (0.86, 2.19) | 0.191 |
| Vasodilators, n (%) |  |  |  |  |  |
| No | 452 (62.87) | 313 (65.34) | 139 (57.92) | Ref | - |
| Yes | 267 (37.13) | 166 (34.66) | 101 (42.08) | 1.37 (1.00, 1.88) | 0.052 |
| Cardiac glycosides, n (%) |  |  |  |  |  |
| No | 695 (96.66) | 464 (96.87) | 231 (96.25) | Ref | - |
| Yes | 24 (3.34) | 15 (3.13) | 9 (3.75) | 1.21 (0.52, 2.80) | 0.664 |
| Antidiabetics, n (%) |  |  |  |  |  |
| No | 585 (81.36) | 405 (84.55) | 180 (75.00) | Ref | - |
| Yes | 134 (18.64) | 74 (15.45) | 60 (25.00) | 1.82 (1.24, 2.68) | 0.002 |
| Anti PD, n (%) |  |  |  |  |  |
| No | 715 (99.44) | 478 (99.79) | 237 (98.75) | Ref | - |
| Yes | 4 (0.56) | 1 (0.21) | 3 (1.25) | 6.05 (0.63, 58.48) | 0.120 |
| Anxiolytics, n (%) |  |  |  |  |  |
| No | 714 (99.30) | 477 (99.58) | 237 (98.75) | Ref | - |
| Yes | 5 (0.70) | 2 (0.42) | 3 (1.25) | 3.02 (0.50, 18.19) | 0.228 |
| Anticholinergics, n (%) |  |  |  |  |  |
| No | 704 (97.91) | 471 (98.33) | 233 (97.08) | Ref | - |
| Yes | 15 (2.09) | 8 (1.67) | 7 (2.92) | 1.77 (0.63, 4.94) | 0.276 |
| AD, n (%) |  |  |  |  |  |
| No | 703 (97.77) | 470 (98.12) | 233 (97.08) | Ref | - |
| Yes | 16 (2.23) | 9 (1.88) | 7 (2.92) | 1.57 (0.58, 4.27) | 0.377 |
| AEDs, n (%) |  |  |  |  |  |
| No | 693 (96.38) | 467 (97.49) | 226 (94.17) | Ref | - |
| Yes | 26 (3.62) | 12 (2.51) | 14 (5.83) | 2.41 (1.10, 5.30) | 0.028 |
| Antipsychotics, n (%) |  |  |  |  |  |
| No | 710 (98.75) | 473 (98.75) | 237 (98.75) | Ref | - |
| Yes | 9 (1.25) | 6 (1.25) | 3 (1.25) | 1.00 (0.25, 4.03) | 0.998 |
| BZDs, n (%) |  |  |  |  |  |
| No | 686 (95.41) | 463 (96.66) | 223 (92.92) | Ref | - |
| Yes | 33 (4.59) | 16 (3.34) | 17 (7.08) | 2.21 (1.09, 4.45) | 0.027 |
| Zdrugs, n (%) |  |  |  |  |  |
| No | 709 (98.61) | 476 (99.37) | 233 (97.08) | Ref | - |
| Yes | 10 (1.39) | 3 (0.63) | 7 (2.92) | 4.77 (1.22, 18.60) | 0.025 |
| Opioids, n (%) |  |  |  |  |  |
| No | 669 (93.05) | 444 (92.69) | 225 (93.75) | Ref | - |
| Yes | 50 (6.95) | 35 (7.31) | 15 (6.25) | 0.85 (0.45, 1.58) | 0.600 |
| **Laboratory indicators** |  |  |  |  |  |
| Alb, n (%) |  |  |  |  |  |
| ≥34 | 422 (58.69) | 299 (62.42) | 123 (51.25) | Ref | - |
| <34 | 297 (41.31) | 180 (37.58) | 117 (48.75) | 1.58 (1.15, 2.16) | 0.004 |
| Hb, Median (Q_1_, Q_3_) | 114.00 (97.00, 126.00) | 114.00 (98.50, 126.14) | 114.00 (95.75, 126.00) | 1.00 (0.99, 1.01) | 0.568 |
| **Other variables** |  |  |  |  |  |
| Sleep duration, Median (Q_1_, Q_3_) | 6.50 (5.50, 7.00) | 6.50 (5.50, 7.00) | 6.50 (5.50, 7.00) | 1.01 (0.89, 1.14) | 0.918 |
| Consciousness, n (%) |  |  |  |  |  |
| Conscious | 706 (98.19) | 469 (97.91) | 237 (98.75) | Ref | - |
| Unconscious | 13 (1.81) | 10 (2.09) | 3 (1.25) | 0.59 (0.16, 2.18) | 0.432 |
| FH-3M, n (%) |  |  |  |  |  |
| No | 660 (91.79) | 455 (94.99) | 205 (85.42) | Ref | - |
| Yes | 59 (8.21) | 24 (5.01) | 35 (14.58) | 3.24 (1.88, 5.58) | <.001 |
| UWA, n (%) |  |  |  |  |  |
| No assistance | 475 (66.06) | 325 (67.85) | 150 (62.5) | Ref | - |
| Wheelchair or bedridden | 192 (26.70) | 134 (27.97) | 58 (24.17) | 0.94 (0.65, 1.35) | 0.729 |
| Support by others or furniture | 10 (1.39) | 2 (0.42) | 8 (3.33) | 8.67 (1.82, 41.31) | 0.007 |
| Walker/crutches/cane | 42 (5.84) | 18 (3.76) | 24 (10.00) | 2.89 (1.52, 5.48) | 0.001 |
| EA, n (%) |  |  |  |  |  |
| No | 701 (97.50) | 477 (99.58) | 224 (93.33) | Ref | - |
| Yes | 18 (2.50) | 2 (0.42) | 16 (6.67) | 17.04 (3.88, 74.72) | <.001 |
| Chaperone, n (%) |  |  |  |  |  |
| Unattended | 36 (5.01) | 27 (5.64) | 9 (3.75) | Ref | - |
| With caregiver | 683 (94.99) | 452 (94.36) | 231 (96.25) | 1.53 (0.71, 3.31) | 0.277 |
| Morse (points), n (%) |  |  |  |  |  |
| <45 | 196 (27.26) | 164 (34.24) | 32 (13.33) | Ref | - |
| ≥45 | 523 (72.74) | 315 (65.76) | 208 (86.67) | 3.38 (2.23, 5.14) | <.001 |
| Pain (points), n (%) |  |  |  |  |  |
| 0 | 570 (79.28) | 380 (79.33) | 190 (79.17) | Ref | - |
| ≥1 | 149 (20.72) | 99 (20.67) | 50 (20.83) | 1.01 (0.69, 1.48) | 0.959 |
| NRS 2002 (points), n (%) |  |  |  |  |  |
| <3 | 409 (56.88) | 283 (59.08) | 126 (52.5) | Ref | - |
| ≥3 | 310 (43.12) | 196 (40.92) | 114 (47.5) | 1.31 (0.96, 1.78) | 0.093 |
| Barthel Index (points), n (%) |  |  |  |  |  |
| 0~20 | 36 (5.01) | 28 (5.85) | 8 (3.33) | Ref | - |
| 21~60 | 172 (23.92) | 104 (21.71) | 68 (28.33) | 2.29 (0.98, 5.32) | 0.054 |
| 61~90 | 322 (44.78) | 203 (42.38) | 119 (49.58) | 2.05 (0.91, 4.65) | 0.085 |
| 91-99 | 79 (10.99) | 52 (10.86) | 27 (11.25) | 1.82 (0.73, 4.53) | 0.200 |
| 100 | 110 (15.30) | 92 (19.21) | 18 (7.50) | 0.68 (0.27, 1.74) | 0.427 |
| Indw Cath, n (%) |  |  |  |  |  |
| 0 | 566 (78.72) | 363 (75.78) | 203 (84.58) | Ref | - |
| 1 | 95 (13.21) | 64 (13.36) | 31 (12.92) | 0.87 (0.55, 1.37) | 0.542 |
| ≥2 | 58 (8.07) | 52 (10.86) | 6 (2.50) | 0.21 (0.09, 0.49) | <.001 |
| Department, n (%) |  |  |  |  |  |
| Department of internal medicine | 464 (64.53) | 297 (62.00) | 167 (69.58) | Ref | - |
| Department of surgery | 184 (25.59) | 139 (29.02) | 45 (18.75) | 0.58 (0.39, 0.85) | 0.005 |
| Department of rehabilitation medicine | 43 (5.98) | 20 (4.18) | 23 (9.58) | 2.05 (1.09, 3.83) | 0.026 |
| Miscellaneous specialties | 28 (3.89) | 23 (4.80) | 5 (2.08) | 0.39 (0.14, 1.04) | 0.059 |

**Supplementary Table S4**

**Table S4. Multivariate stepwise regression analysis of variables.**

| **Variable** | **SR-FS** | | **SR-BS & SR-BE** | |
| --- | --- | --- | --- | --- |
|  | **OR (95% CI)** | ***P*-value** | **OR (95% CI)** | ***P*-value** |
| **Comorbidities** |  |  |  |  |
| Hypothyroidism |  |  |  |  |
| No | Ref | - | Ref | - |
| Yes | 2.30 (0.86, 6.32) | 0.099 | 2.43 (0.91, 6.63) | 0.077 |
| OP |  |  |  |  |
| No | Ref | - | Ref | - |
| Yes | 3.27 (0.84, 14.39) | 0.095 | 3.43 (0.88, 15.11) | 0.082 |
| Dizziness |  |  |  |  |
| No | Ref | - | Ref | - |
| Yes | 4.92 (1.34, 23.60) | 0.024 | 4.80 (1.32, 22.89) | 0.026 |
| Stroke |  |  |  |  |
| No | Ref | - | Ref | - |
| Yes | 1.42 (0.93, 2.15) | 0.100 | 1.49 (0.99, 2.24) | 0.056 |
| Epilepsy |  |  |  |  |
| No | Ref | - | Ref | - |
| Yes | 17.80 (3.46, 134.99) | 0.002 | 18.11 (3.56, 135.3) | 0.002 |
| **M**edications |  |  |  |  |
| Polypharmacy |  |  |  |  |
| No | Ref | - |  |  |
| Yes | 1.18 (0.80, 1.75) | 0.398 |  |  |
| AC |  |  |  |  |
| No | Ref | - | Ref | - |
| Yes | 1.52 (0.91, 2.52) | 0.104 | 1.57 (0.95, 2.58) | 0.076 |
| BZDs |  |  |  |  |
| No | Ref | - | Ref | - |
| Yes | 2.11 (0.95, 4.66) | 0.064 | 2.18 (0.99, 4.78) | 0.051 |
| Zdrugs |  |  |  |  |
| No |  |  | Ref | - |
| Yes |  |  | 2.96 (0.68, 15.29) | 0.158 |
| **Laboratory indicators** |  |  |  |  |
| Alb |  |  |  |  |
| ≥34 | Ref | - | Ref | - |
| <34 | 1.63 (1.13, 2.36) | 0.009 | 1.64 (1.13, 2.36) | 0.009 |
| **Other variables** |  |  |  |  |
| FH-3M |  |  |  |  |
| No | Ref | - | Ref | - |
| Yes | 2.23 (1.19, 4.24) | 0.013 | 2.15 (1.14, 4.10) | 0.018 |
| UWA |  |  |  |  |
| No assistance | Ref | - | Ref | - |
| Wheelchair or bedridden | 0.53 (0.32, 0.86) | 0.011 | 0.54 (0.33, 0.88) | 0.015 |
| Support by others or furniture | 7.47(1.60, 54.14) | 0.019 | 7.68 (1.64, 55.76) | 0.018 |
| Walker/crutches/cane | 1.41 (0.68, 2.92) | 0.359 | 1.42 (0.68, 2.95) | 0.344 |
| EA |  |  |  |  |
| No | Ref | - | Ref | - |
| Yes | 9.99 (2.61, 65.98) | 0.003 | 9.97 (2.58, 66.15) | 0.003 |
| Morse (points) |  |  |  |  |
| <45 | Ref | - | Ref | - |
| ≥45 | 2.45 (1.53, 4.02) | <0.001 | 2.51 (1.58, 4.07) | <0.001 |
| Barthel Index (points) |  |  |  |  |
| 0~20 | Ref | - | Ref | - |
| 21~60 | 2.55 (0.95, 7.70) | 0.077 | 2.71 (1.00, 8.21) | 0.061 |
| 61~90 | 1.78 (0.65, 5.43) | 0.284 | 1.91 (0.69, 5.83) | 0.232 |
| 91-99 | 2.14 (0.69, 7.25) | 0.201 | 2.31 (0.74, 7.83) | 0.162 |
| 100 | 0.97 (0.31, 3.33) | 0.961 | 1.06 (0.33, 3.65) | 0.922 |
| Indw Cath |  |  |  |  |
| 0 | Ref | - | Ref | - |
| 1 | 0.79 (0.46, 1.32) | 0.377 | 0.79 (0.46, 1.32) | 0.368 |
| ≥2 | 0.18 (0.06, 0.46) | <0.001 | 0.18 (0.06, 0.45) | <0.001 |

**Supplementary Table S5**

**Table S5. Clinical features of patients in the training and testing set.**

| **Variables** | **Training set (n = 719)** | **Testing set (n = 307)** | ***P*-value** |
| --- | --- | --- | --- |
|  |  |  | 1.000 |
| Non fall | 479 (66.62) | 205 (66.78) |  |
| Fall | 240 (33.38) | 102 (33.22) |  |
| **Demographic characteristics** |  |  |  |
| Age (years), n (%) |  |  | 0.845 |
| 60~69 | 291 (40.47) | 128 (41.69) |  |
| 70~79 | 286 (39.78) | 123 (40.07) |  |
| ≥80 | 142 (19.75) | 56 (18.24) |  |
| Gender, n (%) |  |  | 0.961 |
| Male | 401 (55.77) | 170 (55.37) |  |
| Female | 318 (44.23) | 137 (44.63) |  |
| Marital status, n (%) |  |  |  |
| Married |  |  | 0.542 |
| Non married | 603 (83.87) | 252 (82.08) |  |
| Education, n (%) | 116 (16.13) | 55 (17.92) |  |
| Primary education or below |  |  | 0.788 |
| High school or vocational secondary school | 436 (60.64) | 191 (62.21) |  |
| Tertiary education or above | 233 (32.41) | 93 (30.29) |  |
| **Comorbidities** |  |  |  |
| Bp, n (%) |  |  | 0.624 |
| No | 354 (49.24) | 157 (51.14) |  |
| Yes | 365 (50.76) | 150 (48.86) |  |
| Diabetes, n (%) |  |  | 0.445 |
| No | 538 (74.83) | 222 (72.31) |  |
| Yes | 181 (25.17) | 85 (27.69) |  |
| CHD, n (%) |  |  | 0.915 |
| No | 603 (83.87) | 259 (84.36) |  |
| Yes | 116 (16.13) | 48 (15.64) |  |
| COPD, n (%) |  |  | 1.000 |
| No | 694 (96.52) | 297 (96.74) |  |
| Yes | 25 (3.48) | 10 (3.26) |  |
| CKD, n (%) |  |  | 0.912 |
| No | 681 (94.71) | 292 (95.11) |  |
| Yes | 38 (5.29) | 15 (4.89) |  |
| HF, n (%) |  |  | 0.305 |
| No | 683 (94.99) | 286 (93.16) |  |
| Yes | 36 (5.01) | 21 (6.84) |  |
| Dyslipidemia, n (%) |  |  | 0.073 |
| No | 694 (96.52) | 288 (93.81) |  |
| Yes | 25 (3.48) | 19 (6.19) |  |
| Hypothyroidism, n (%) |  |  | 0.259 |
| No | 696 (96.8) | 292 (95.11) |  |
| Yes | 23 (3.2) | 15 (4.89) |  |
| Arrhythmia, n (%) |  |  | 0.121 |
| No | 583 (81.08) | 262 (85.34) |  |
| Yes | 136 (18.92) | 45 (14.66) |  |
| CA, n (%) |  |  | 0.004 |
| No | 442 (61.47) | 218 (71.01) |  |
| Yes | 277 (38.53) | 89 (28.99) |  |
| CI, n (%) |  |  | 0.448 |
| No | 712 (99.03) | 306 (99.67) |  |
| Yes | 7 (0.97) | 1 (0.33) |  |
| PD, n (%) |  |  | 1.000 |
| No | 711 (98.89) | 304 (99.02) |  |
| Yes | 8 (1.11) | 3 (0.98) |  |
| DSD, n (%) |  |  | 0.668 |
| No | 692 (96.24) | 293 (95.44) |  |
| Yes | 27 (3.76) | 14 (4.56) |  |
| OP, n (%) |  |  | 0.170 |
| No | 707 (98.33) | 297 (96.74) |  |
| Yes | 12 (1.67) | 10 (3.26) |  |
| Dizziness, n (%) |  |  | 0.925 |
| No | 705 (98.05) | 302 (98.37) |  |
| Yes | 14 (1.95) | 5 (1.63) |  |
| Hypotension, n (%) |  |  | 0.187 |
| No | 713 (99.17) | 307 (100) |  |
| Yes | 6 (0.83) | 0 (0) |  |
| Stroke, n (%) |  |  | 0.712 |
| No | 525 (73.02) | 220 (71.66) |  |
| Yes | 194 (26.98) | 87 (28.34) |  |
| RD, n (%) |  |  | 0.881 |
| No | 671 (93.32) | 285 (92.83) |  |
| Yes | 48 (6.68) | 22 (7.17) |  |
| Epilepsy, n (%) |  |  | 1.000 |
| No | 706 (98.19) | 302 (98.37) |  |
| Yes | 13 (1.81) | 5 (1.63) |  |
| GA, n (%) |  |  | 0.015 |
| No | 662 (92.07) | 296 (96.42) |  |
| Yes | 57 (7.93) | 11 (3.58) |  |
| VI, n (%) |  |  | 0.560 |
| No | 325 (45.2) | 132 (43) |  |
| Yes | 394 (54.8) | 175 (57) |  |
| HI, n (%) |  |  | 0.912 |
| No | 503 (69.96) | 213 (69.38) |  |
| Yes | 216 (30.04) | 94 (30.62) |  |
| Sleep disorder, n (%) |  |  | 0.073 |
| No | 585 (81.36) | 234 (76.22) |  |
| Yes | 134 (18.64) | 73 (23.78) |  |
| **Medications** |  |  |  |
| Polypharmacy, n (%) |  |  | 0.807 |
| No | 461 (64.12) | 200 (65.15) |  |
| Yes | 258 (35.88) | 107 (34.85) |  |
| AC, n (%) |  |  | 0.120 |
| No | 617 (85.81) | 251 (81.76) |  |
| Yes | 102 (14.19) | 56 (18.24) |  |
| AP, n (%) |  |  | 0.286 |
| No | 600 (83.45) | 247 (80.46) |  |
| Yes | 119 (16.55) | 60 (19.54) |  |
| PPI, n (%) |  |  | 1.000 |
| No | 491 (68.29) | 210 (68.4) |  |
| Yes | 228 (31.71) | 97 (31.6) |  |
| Diuretics, n (%) |  |  | 0.885 |
| No | 562 (78.16) | 238 (77.52) |  |
| Yes | 157 (21.84) | 69 (22.48) |  |
| NSAIDs, n (%) |  |  | 0.427 |
| No | 673 (93.6) | 292 (95.11) |  |
| Yes | 46 (6.4) | 15 (4.89) |  |
| MMR, n (%) |  |  | 0.330 |
| No | 714 (99.3) | 307 (100) |  |
| Yes | 5 (0.7) | 0 (0) |  |
| Statins, n (%) |  |  | 0.118 |
| No | 572 (79.55) | 230 (74.92) |  |
| Yes | 147 (20.45) | 77 (25.08) |  |
| α blockers, n (%) |  |  | 0.710 |
| No | 683 (94.99) | 294 (95.77) |  |
| Yes | 36 (5.01) | 13 (4.23) |  |
| β blockers, n (%) |  |  | 0.572 |
| No | 636 (88.46) | 267 (86.97) |  |
| Yes | 83 (11.54) | 40 (13.03) |  |
| Vasodilators, n (%) |  |  | 1.000 |
| No | 452 (62.87) | 193 (62.87) |  |
| Yes | 267 (37.13) | 114 (37.13) |  |
| Cardiac glycosides, n (%) |  |  | 0.479 |
| No | 695 (96.66) | 300 (97.72) |  |
| Yes | 24 (3.34) | 7 (2.28) |  |
| Antidiabetics, n (%) |  |  | 0.148 |
| No | 585 (81.36) | 237 (77.2) |  |
| Yes | 134 (18.64) | 70 (22.8) |  |
| Anti PD, n (%) |  |  | 0.433 |
| No | 715 (99.44) | 304 (99.02) |  |
| Yes | 4 (0.56) | 3 (0.98) |  |
| Anxiolytics, n (%) |  |  | 0.176 |
| No | 714 (99.3) | 302 (98.37) |  |
| Yes | 5 (0.7) | 5 (1.63) |  |
| Anticholinergics, n (%) |  |  | 0.085 |
| No | 704 (97.91) | 294 (95.77) |  |
| Yes | 15 (2.09) | 13 (4.23) |  |
| AD, n (%) |  |  | 0.055 |
| No | 703 (97.77) | 306 (99.67) |  |
| Yes | 16 (2.23) | 1 (0.33) |  |
| AEDs, n (%) |  |  | 0.963 |
| No | 693 (96.38) | 295 (96.09) |  |
| Yes | 26 (3.62) | 12 (3.91) |  |
| Antipsychotics, n (%) |  |  | 0.401 |
| No | 710 (98.75) | 301 (98.05) |  |
| Yes | 9 (1.25) | 6 (1.95) |  |
| BZDs, n (%) |  |  | 0.965 |
| No | 686 (95.41) | 292 (95.11) |  |
| Yes | 33 (4.59) | 15 (4.89) |  |
| Zdrugs, n (%) |  |  | 0.765 |
| No | 709 (98.61) | 304 (99.02) |  |
| Yes | 10 (1.39) | 3 (0.98) |  |
| Opioids, n (%) |  |  | 0.755 |
| No | 669 (93.05) | 288 (93.81) |  |
| Yes | 50 (6.95) | 19 (6.19) |  |
| **Laboratory indicators** |  |  |  |
| Alb, n (%) |  |  | 0.763 |
| ≥34 | 422 (58.69) | 184 (59.93) |  |
| <34 | 297 (41.31) | 123 (40.07) |  |
| Hb, Median (Q_1_, Q_3_) | 114 (97, 126) | 116 (98.5, 127) | 0.335 |
| **Other variables** |  |  |  |
| Sleep duration, Median (Q_1_, Q_3_) | 6.5 (5.5, 7) | 6.5 (5.5, 7) | 0.063 |
| Consciousness, n (%) |  |  | 0.134 |
| Conscious | 706 (98.19) | 296 (96.42) |  |
| Unconscious | 13 (1.81) | 11 (3.58) |  |
| FH-3M, n (%) |  |  | 0.987 |
| No | 660 (91.79) | 281 (91.53) |  |
| Yes | 59 (8.21) | 26 (8.47) |  |
| UWA, n (%) |  |  | 0.084 |
| No assistance | 475 (66.06) | 179 (58.31) |  |
| Wheelchair or bedridden | 192 (26.7) | 100 (32.57) |  |
| Support by others or furniture | 10 (1.39) | 3 (0.98) |  |
| Walker/crutches/cane | 42 (5.84) | 25 (8.14) |  |
| EA, n (%) |  |  | 0.639 |
| No | 701 (97.5) | 297 (96.74) |  |
| Yes | 18 (2.5) | 10 (3.26) |  |
| Chaperone, n (%) |  |  | 1.000 |
| Unattended | 36 (5.01) | 15 (4.89) |  |
| With caregiver | 683 (94.99) | 292 (95.11) |  |
| MFS (points), n (%) |  |  | 0.419 |
| <45 | 196 (27.26) | 92 (29.97) |  |
| ≥45 | 523 (72.74) | 215 (70.03) |  |
| NRS (points), n (%) |  |  | 0.315 |
| 0 | 570 (79.28) | 234 (76.22) |  |
| ≥1 | 149 (20.72) | 73 (23.78) |  |
| NRS 2002 (points), n (%) |  |  | 0.220 |
| <3 | 409 (56.88) | 188 (61.24) |  |
| ≥3 | 310 (43.12) | 119 (38.76) |  |
| mBI (points), n (%) |  |  | 0.238 |
| 0~20 | 36 (5.01) | 13 (4.23) |  |
| 21~60 | 172 (23.92) | 93 (30.29) |  |
| 61~90 | 322 (44.78) | 121 (39.41) |  |
| 91-99 | 79 (10.99) | 30 (9.77) |  |
| 100 | 110 (15.3) | 50 (16.29) |  |
| Indw Cath, n (%) |  |  | 0.810 |
| 0 | 566 (78.72) | 247 (80.46) |  |
| 1 | 95 (13.21) | 38 (12.38) |  |
| ≥2 | 58 (8.07) | 22 (7.17) |  |
| Department, n (%) |  |  | < 0.001 |
| Department of internal medicine | 464 (64.53) | 175 (57) |  |
| Department of surgery | 184 (25.59) | 117 (38.11) |  |
| Department of rehabilitation medicine | 43 (5.98) | 7 (2.28) |  |
| Miscellaneous specialties | 28 (3.89) | 8 (2.61) |  |

**Supplementary Figure S1**

**
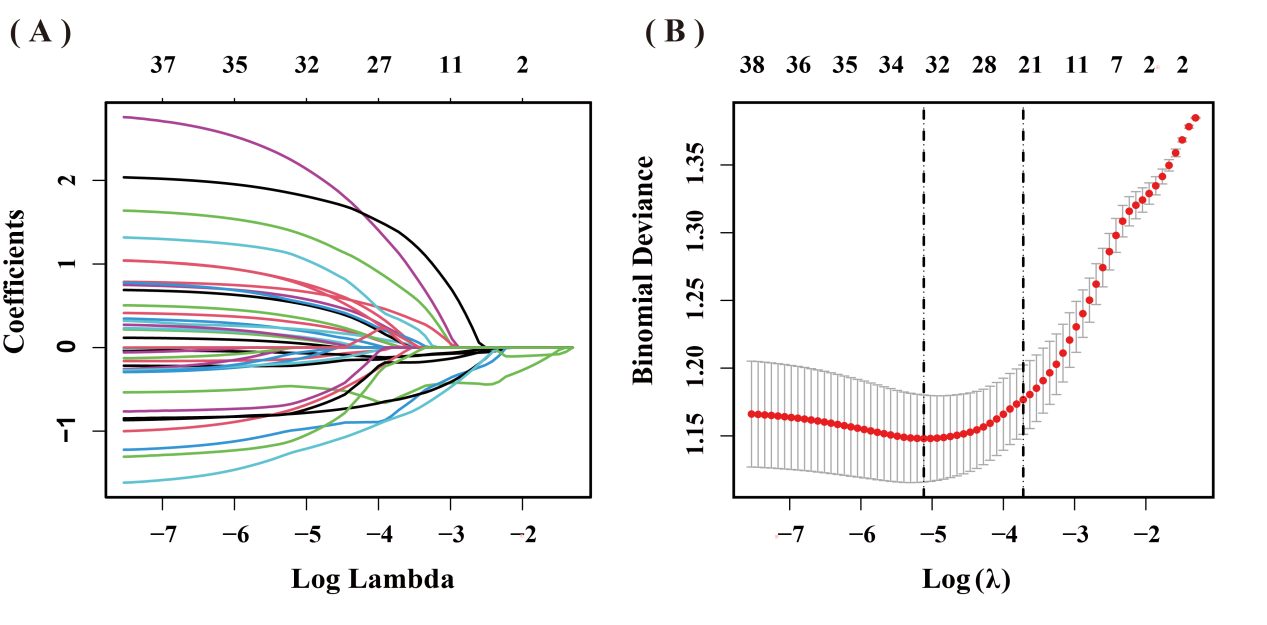
**

**Figure S1. LASSO regression analysis plot.** (A) Cross-validation plot for penalty parameters (λ). (B) Regression coefficient plot for predictor variables with different penalty parameters (λ).

**Supplementary Figure S2**

**
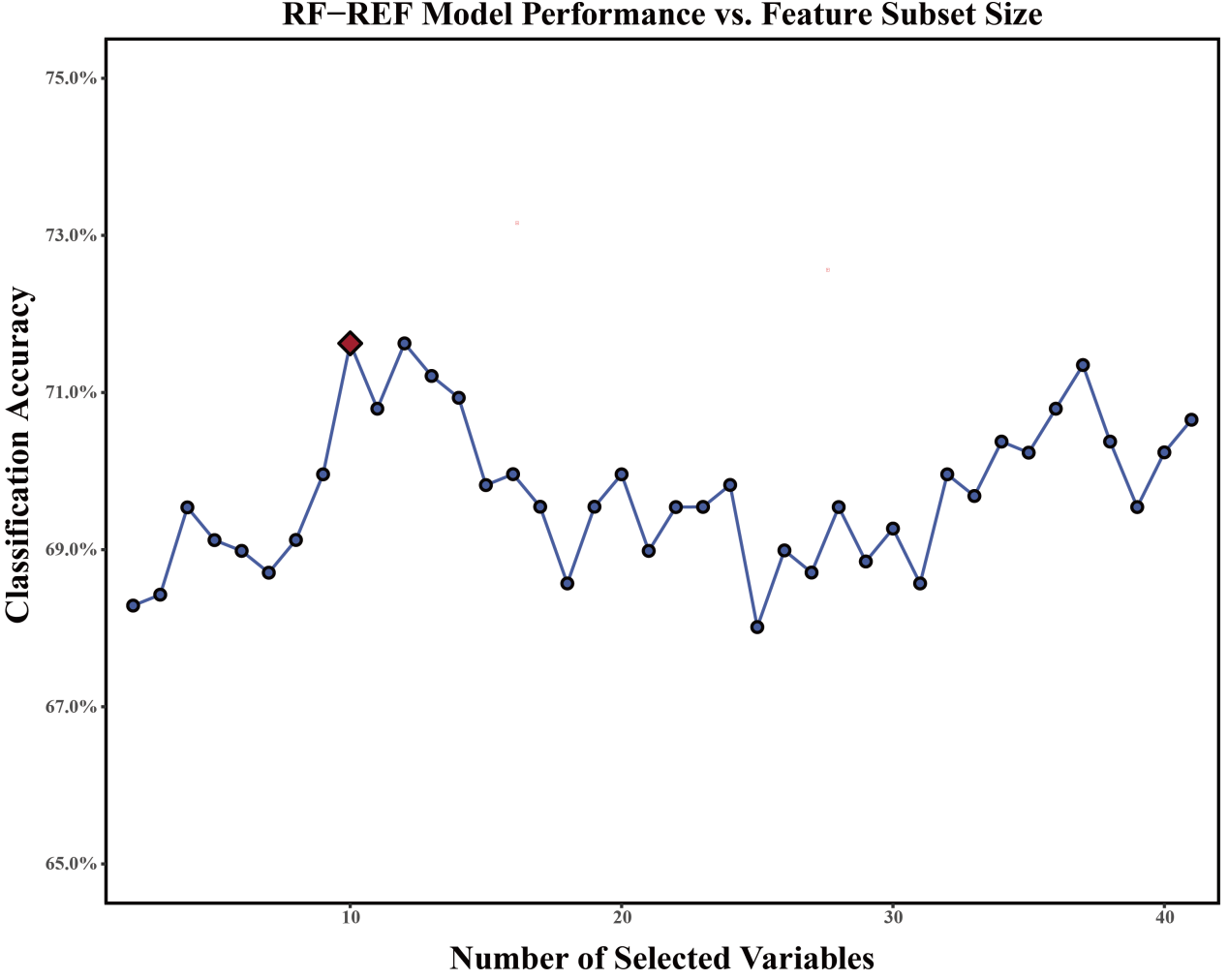
**

**Figure S2. RF-REF model performance varies with feature subset size.**
